# β-Sitosterol–Naringenin Co-Loaded Nanophytosomes: Encapsulation, Compound-Specific Release Kinetics, Machine Learning-Assisted Release Prediction, and Cell-Dependent Anticancer Activity

**DOI:** 10.3390/ijms27219494

**Published:** 2026-10-24

**Authors:** Tabarek H. Mahmood, Ali Al-Samydai, Firas F. Al-Mamoori, Moath Alqaraleh, Maha N. Abu Hajleh, Amal Mayyas, Said Moshawih, Alaa Al-Sanabrah, Qout Qtashat, Rawan AlMafrachi, Hussam Hussain Kassim, Ahmed S. A. Ali Agha, Hany Akeel Al-Hussaniy, Walhan AlShaer

**Affiliations:** 1 College of Dentistry, Ibn Sina University of Medical and Pharmaceutical Sciences, Baghdad 10015, Iraq; tabarek.h.m@ibnsina.edu.iq; 2 Pharmacological and Diagnostic Research Center, Faculty of Pharmacy, Al-Ahliyya Amman University, Amman 19328, Jordan; s.moshawih@ammanu.edu.jo (S.M.); a.sanabrah@ammanu.edu.jo (A.A.-S.); q.qtashat@ammanu.edu.jo (Q.Q.); rawanalmafrachi@gmail.com (R.A.); hhk699@gmail.com (H.H.K.); asat3u@gmail.com (A.S.A.A.A.); 3 School of Pharmaceutical Science, University of Babylon, Babylon 51001, Iraq; phar.firas.falah@uobabylon.edu.iq; 4 Department of Medical Laboratory Sciences, Faculty of Allied Medical Sciences, Al-Balqa Applied University, Al-Salt 19117, Jordan; muath.garalleh@bau.edu.jo; 5 Department of Cosmetic Science, Pharmacological and Diagnostic Research Centre, Faculty of Allied Medical Sciences, Al-Ahliyya Amman University, Amman 19328, Jordan; m.abuhajleh@ammanu.edu.jo; 6 Faculty of Health Sciences, Department of Pharmacy, American University of Madaba, Madaba 11821, Jordan; a.mayyas@aum.edu.jo; 7 Department of Pharmaceutical Sciences, School of Pharmacy, The University of Jordan, Amman 11942, Jordan; 8 Department of Pharmacology, College of Pharmacy, Al-Nisour University, Baghdad 10013, Iraq; hany_akeel2000@yahoo.com; 9 Cell Therapy Centre, The University of Jordan, Amman 11942, Jordan; walhan.alshaer@ju.edu.jo

**Keywords:** β-Sitosterol, naringenin, nanophytosomes, co-delivery, drug-release kinetics, machine learning, molecular docking, cancer nanomedicine

## Abstract

β-Sitosterol and naringenin have anticancer potential but poor biopharmaceutical properties, while their behavior when co-encapsulated remains poorly defined. This study examined whether co-loading alters encapsulation efficiency, compound-specific release behavior, predictability, and cell-dependent activity. Single- and co-loaded nanophytosomes were characterized for encapsulation and release. Release profiles underwent comparative kinetic modelling, uncertainty-aware augmentation, early-to-late ridge-regression forecasting, Weibull-parameter reconstruction, and exploratory reconstructed-replicate holdout assessment. Cytotoxicity was assessed in A549 (ATCC CCL-185), HCT116 (ATCC CCL-247), and HEK293 (ATCC CRL-1573) cells, with docking against MEK1, COX-2, and PI3Kα. Co-loading increased β-sitosterol encapsulation from 5.92% to 66.61% but reduced naringenin encapsulation from 89.76% to 40.40% (*p* = 0.02), the co-loaded formulation also exhibited the smallest vesicles (132.7 ± 3.2 nm). At 48 h, release reached 87.30% and 73.24%, respectively; Weibull best described β-sitosterol and Higuchi naringenin, revealing asynchronous release and a time-dependent delivery ratio. Across 1200 augmented profiles, first-4-h measurements predicted 6–48-h release with R^2^ = 0.976–0.996, while parameter-based reconstruction reproduced compound-specific trajectories. Exploratory reconstructed-replicate assessment yielded a pooled R^2^ of 0.938 (mean absolute error (MAE) = 3.32%; root-mean-square error (RMSE) = 4.78%). The co-loaded formulation showed its strongest relative activity in A549 cells and better preserved HEK293 viability than the free combination, whereas the free combination was more active in HCT116 cells. Naringenin docked across all targets (7.58 to −8.40 kcal/mol), whereas β-sitosterol showed weaker binding and no COX-2 pose. Co-loading was associated with opposing encapsulation trends, while the resulting formulation exhibited compound-specific release kinetics and cell-dependent responses rather than uniformly enhanced activity. Integrating physicochemical characterization, kinetic discrimination, predictive modelling, docking, and cell testing provides a multidimensional framework for dual-bioactive nanophytosomes.

## 1. Introduction

β-Sitosterol and naringenin are structurally distinct natural bioactives with reported anticancer properties but physicochemical limitations that can restrict their formulation and delivery. β-Sitosterol is a hydrophobic phytosterol with antiproliferative and pro-apoptotic activity in experimental cancer models [1]. Naringenin, the citrus-derived flavanone aglycone of naringin, modulates cancer-cell proliferation, apoptosis, and cell-cycle regulation [2]. These properties are relevant to lung and colorectal cancers, which remain among the leading contributors to global cancer incidence and mortality [3]. Despite advances in cancer treatment, limited tumour selectivity and dose-limiting toxicity continue to motivate the development of delivery strategies capable of modifying drug exposure and cellular effects [4,5,6].

Nanocarrier-based drug-delivery systems offer opportunities to improve the formulation of poorly soluble compounds and modulate their release characteristics [7,8,9]. Recent advances in cancer nanomedicine further illustrate their broader therapeutic applications, including controlled delivery of STING-pathway agonists [10] and nanoplatforms designed to coordinate immunogenic cell death, antigen presentation, and tumor-microenvironment modulation [11]. Against this broader background, the poor aqueous solubility and suboptimal biopharmaceutical properties of β-sitosterol and naringenin support their incorporation into phospholipid-based nanocarriers [7,8,9].

The behavior of multicomponent phospholipid carriers cannot necessarily be predicted from their corresponding single-loaded systems. When chemically distinct compounds are co-loaded, compound–compound and compound–lipid interactions, together with differences in molecular structure and hydrophobicity, may influence the retention and release of each component. Consequently, simultaneous incorporation may alter encapsulation efficiency and generate distinct release kinetics, resulting in asynchronous delivery and a time-dependent release ratio. Quantitative encapsulation measurements and time-resolved release analysis are therefore required to establish each compound’s formulation behavior.

Kinetic modelling provides a quantitative means of characterizing these release profiles. Comparative evaluation of empirical release models can distinguish compound-specific temporal behavior [12,13], while machine learning approaches can complement conventional kinetic analysis by predicting later release from early experimental observations and reconstructing release profiles from estimated kinetic parameters [14]. Integrating experimental measurements, kinetic-model discrimination, and predictive modelling therefore provides complementary characterization of multicomponent formulations by establishing their release behavior, describing its temporal pattern, and evaluating its predictability.

The functional consequences of these formulation-dependent differences are also relevant because altered encapsulation and release may not translate into equivalent biological effects across different cell types. Nanoformulation can modify the cellular activity of poorly soluble bioactives [15,16], while β-sitosterol and naringenin have demonstrated antiproliferative activity in experimental lung and colorectal cancer models [17,18,19,20].

Recent anticancer investigations have similarly employed malignant and nonmalignant cellular models, including A549 and MRC-5 cells, to evaluate cytotoxicity and selectivity [21]. Such comparative evaluation is relevant to determining whether formulation-associated physicochemical differences are accompanied by cell-dependent biological responses.

Accordingly, this study developed single- and co-loaded β-sitosterol–naringenin nanophytosomes to determine how co-loading affects compound encapsulation and experimentally measured temporal release. Comparative kinetic modelling was integrated with machine learning-based direct prediction and parameter-based profile reconstruction to evaluate the internal predictability of later release from early measurements, while an additional exploratory LOBO analysis was used to assess predictive robustness when complete reconstructed experimental batch trajectories were withheld from model development. Finally, we evaluated free and nanoformulated compounds in A549 lung adenocarcinoma, HCT116 colorectal carcinoma, and HEK293 cells to determine how formulation and co-loading influence cell-dependent cytotoxic responses.

## 2. Results

### 2.1. HPLC Quantification of β-Sitosterol and Naringenin

**Figure 1:**
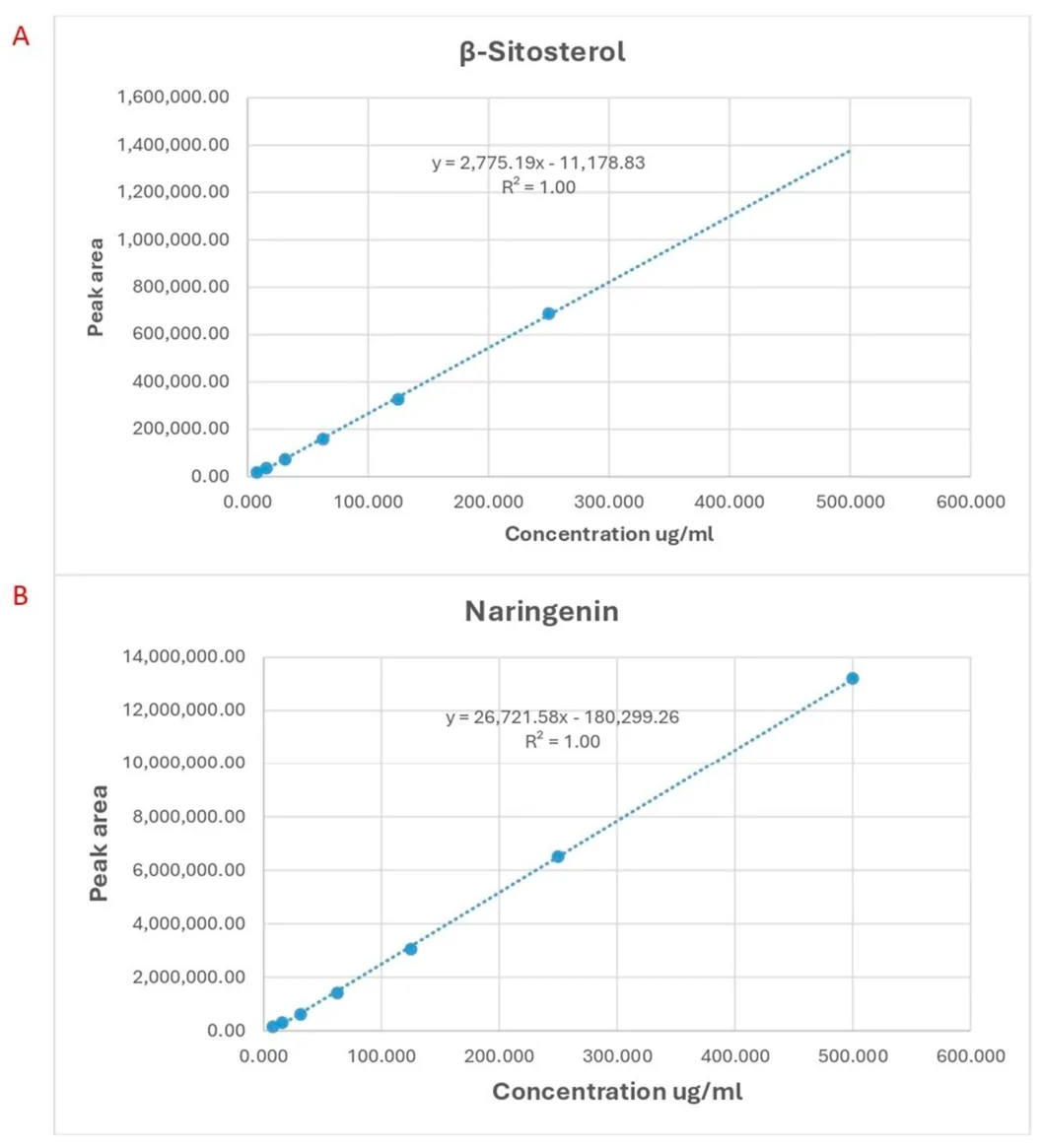
HPLC calibration curves of (**A**) β-sitosterol and (**B**) naringenin, showing the relationship between analyte concentration (µg/mL) and chromatographic peak area.

Calibration curves were constructed for β-sitosterol and naringenin using standard solutions covering concentration ranges up to 250 and 500 µg/mL, respectively. Both analytes demonstrated excellent linearity between concentration and HPLC peak area, with regression equations of y = 2775.2x − 11,179 (R^2^ = 0.9994) for β-sitosterol and y = 26,722x − 180,299 (R^2^ = 0.9997) for naringenin, as presented in Figure 1A,B.

### 2.2. Encapsulation Efficiency of β-Sitosterol and Naringenin

**Figure 2:**
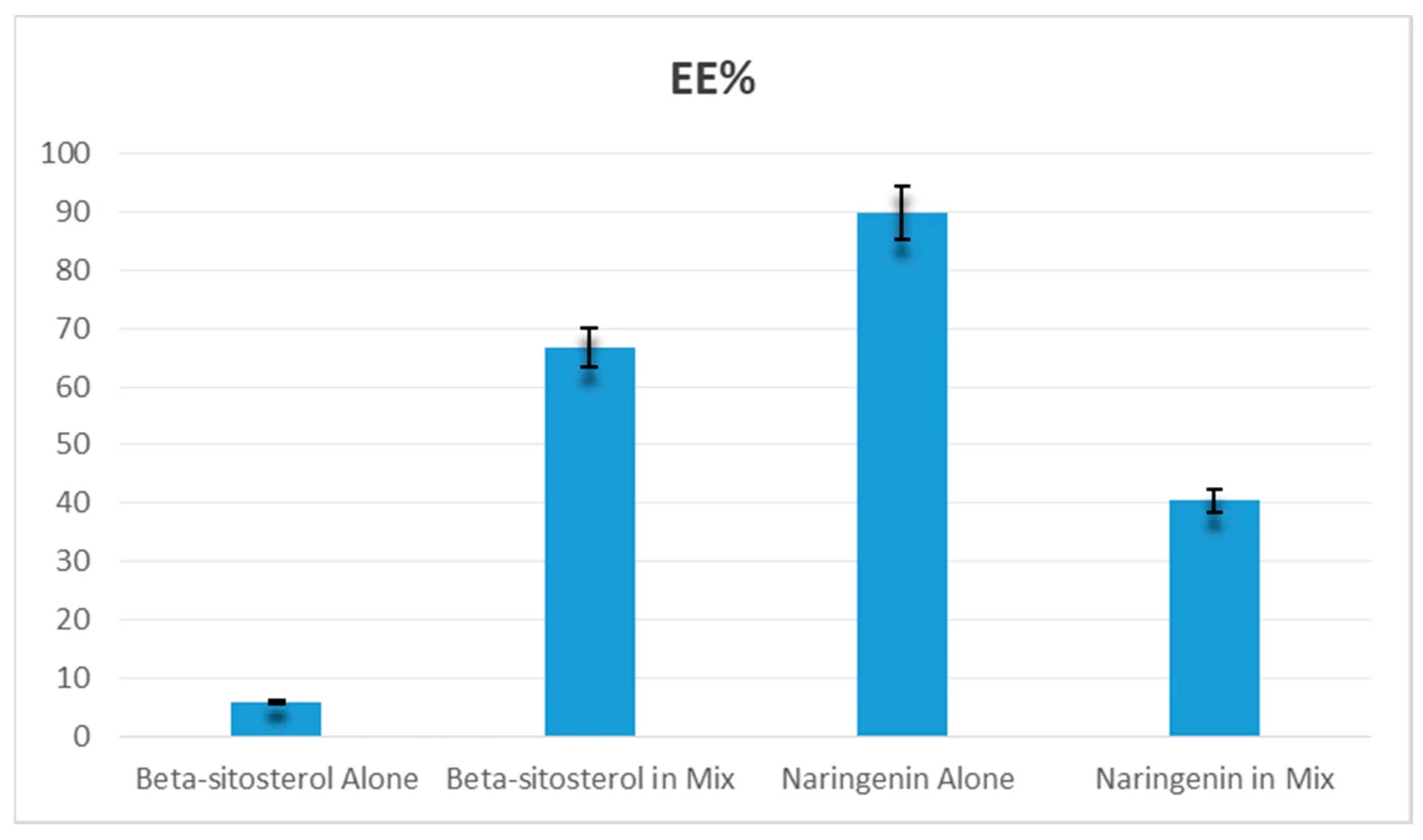
Encapsulation efficiencies of β-sitosterol and naringenin in the single-compound and co-loaded nanophytosomal formulations. Data are presented as mean ± SD.

The encapsulation efficiencies of β-sitosterol and naringenin differed between the single-compound and co-loaded nanophytosomal formulations (Figure 2). The encapsulation efficiency of β-sitosterol increased from 5.92 ± 0.05% in the single-compound formulation to 66.61 ± 7.62% in the co-loaded formulation. In contrast, the encapsulation efficiency of naringenin decreased from 89.76 ± 0.36% in the single-compound formulation to 40.40 ± 0.05% in the co-loaded formulation. The overall difference among the formulations was statistically significant (*p* = 0.02).

### 2.3. Physicochemical and Morphological Characterization of Nanophytosomes

The physicochemical characteristics of the single- and co-loaded nanophytosomal formulations were evaluated in terms of particle size, polydispersity index (PDI), and zeta potential to determine whether incorporation of β-sitosterol and naringenin, individually or in combination, altered the physical properties of the vesicular systems.

**Figure 3:**
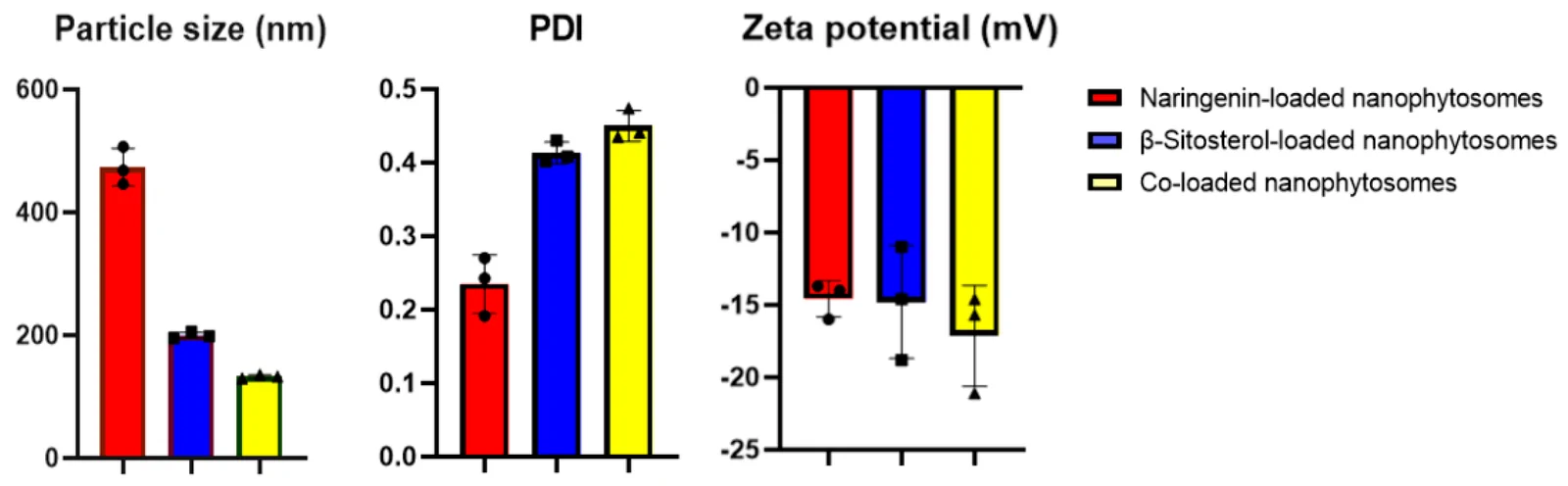
Physicochemical characterization of naringenin-loaded, β-sitosterol-loaded, and co-loaded nanophytosomes. Particle size, polydispersity index (PDI), and zeta potential are shown as individual experimental observations with mean ± SD (n = 3 per formulation).

Particle size differed significantly among the three formulations (one-way ANOVA, *p* < 0.001). Naringenin-loaded nanophytosomes exhibited the largest mean particle size (473.8 ± 29.0 nm, *n* = 3), followed by β-sitosterol-loaded nanophytosomes (199.6 ± 5.3 nm, *n* = 3), whereas the co-loaded formulation exhibited the smallest mean particle size (132.7 ± 3.2 nm, *n* = 3). Pairwise comparisons showed that naringenin-loaded nanophytosomes were significantly larger than both β-sitosterol-loaded and co-loaded formulations (*p* < 0.001), while the co-loaded formulation was also significantly smaller than the β-sitosterol-loaded formulation (*p* < 0.05) (Figure 3). Thus, co-loading was associated with a marked reduction in mean vesicle size relative to either single-loaded formulation.

PDI also differed significantly among formulations (one-way ANOVA, *p* < 0.001). Naringenin-loaded nanophytosomes exhibited the lowest PDI (0.235 ± 0.044, *n* = 3), significantly lower than those of β-sitosterol-loaded (0.413 ± 0.015, *n* = 3) and co-loaded nanophytosomes (0.450 ± 0.021, *n* = 3; both *p* < 0.001). No significant difference was observed between β-sitosterol-loaded and co-loaded formulations (*p* > 0.05) (Figure 3). Accordingly, although co-loading produced the smallest mean vesicle size, the naringenin-loaded formulation exhibited the narrowest particle-size distribution, whereas the two β-sitosterol-containing formulations showed comparatively broader distributions.

All formulations exhibited negative zeta potentials. Mean values were −14.57 ± 1.25 mV for naringenin-loaded nanophytosomes, −14.80 ± 3.90 mV for β-sitosterol-loaded nanophytosomes, and −17.13 ± 3.48 mV for the co-loaded formulation. No significant difference in zeta potential was detected among the formulations (one-way ANOVA, *F*(2,6) = 0.627, *p* = 0.566), indicating that the pronounced formulation-dependent differences in particle size and PDI were not accompanied by a statistically detectable alteration in surface charge.

**Figure 4:**
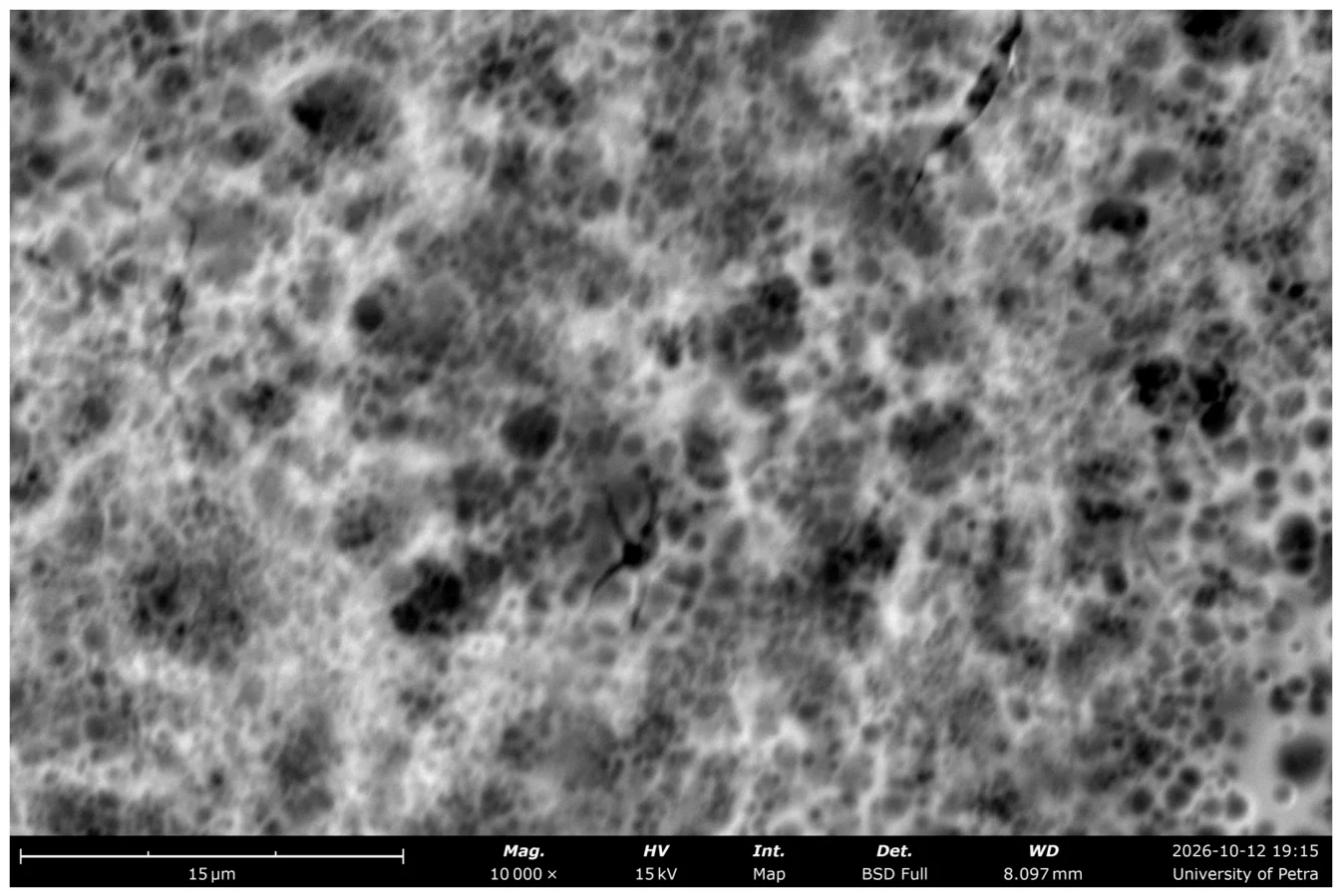
Scanning electron microscopy (SEM) micrograph of lyophilized β-sitosterol–naringenin co-loaded nanophytosomes (10,000×, 15 kV, BSD detector, scale bar = 15 µm).

Scanning electron microscopy (SEM) analysis of the lyophilized β-sitosterol–naringenin co-loaded nanophytosomes showed rounded, quasi-spherical vesicular structures with a porous surface texture (Figure 4). These observations complemented the DLS-based physicochemical characterization by providing morphological information on the dried formulations.

### 2.4. In Vitro Drug Release and Computational Analysis

#### 2.4.1. Experimental Drug-Release Profiles

**Figure 5:**
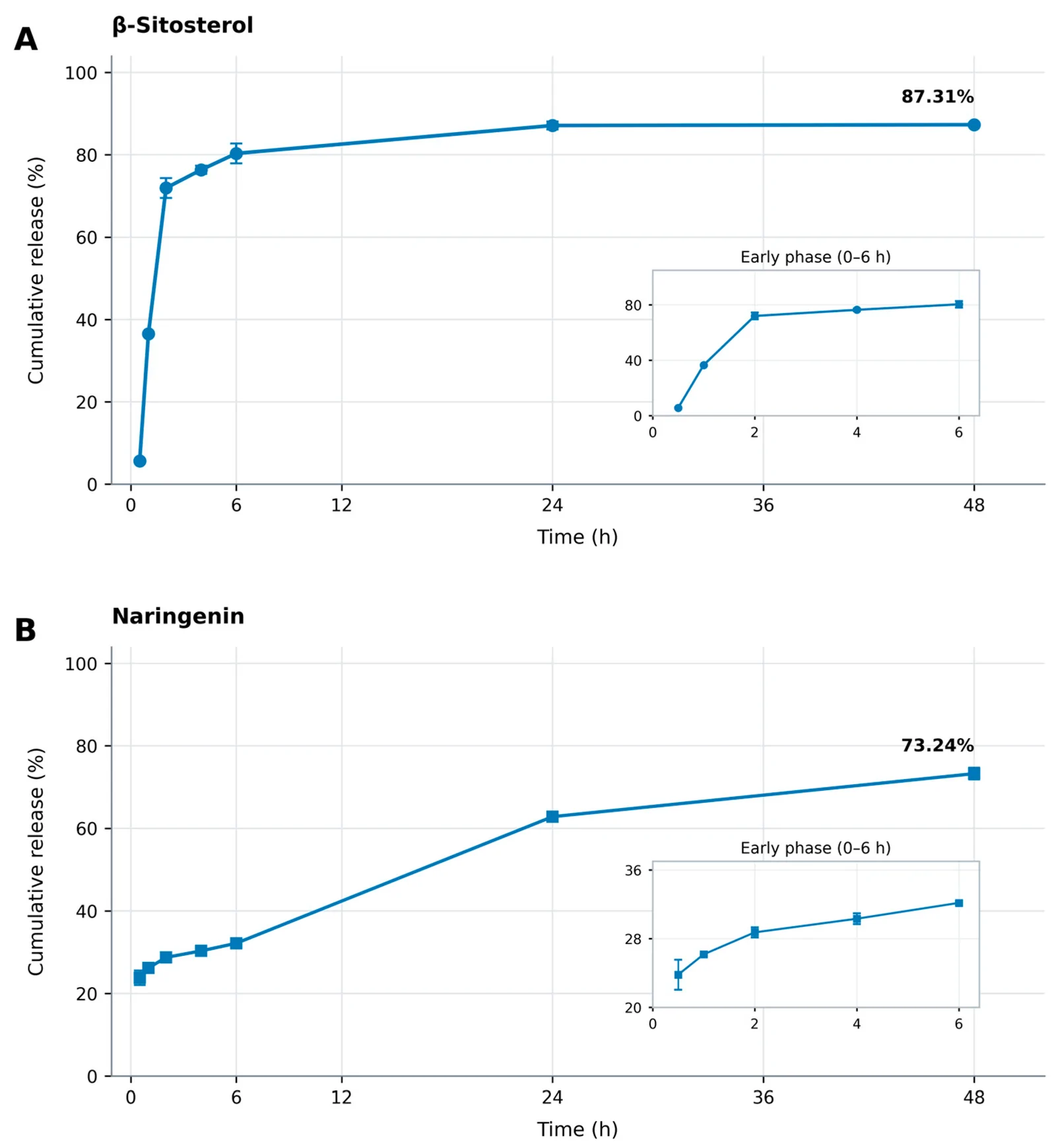
Cumulative in vitro release profiles of (**A**) β-sitosterol and (**B**) naringenin from the co-loaded nanophytosomal formulation. Data are presented as mean ± SD (*n* = 2) at seven experimentally sampled time points. The right-hand panels provide an enlarged view of the initial 0–6 h release phase. Lines connect experimental observations without kinetic-model fitting.

The cumulative release profiles of β-sitosterol and naringenin from the co-loaded nanophytosomal formulation are presented in Figure 5. β-Sitosterol exhibited an initial cumulative release of 5.66% after 30 min, followed by an increase to 87.30% after 48 h. Naringenin showed an initial cumulative release of 23.80% after 30 min and 28.73% after 2 h, increasing gradually to 73.24% after 48 h. Thus, the two compounds exhibited distinct release profiles from the co-loaded formulation.

#### 2.4.2. Generation and Evaluation of Augmented Release Profiles

**Figure 6:**
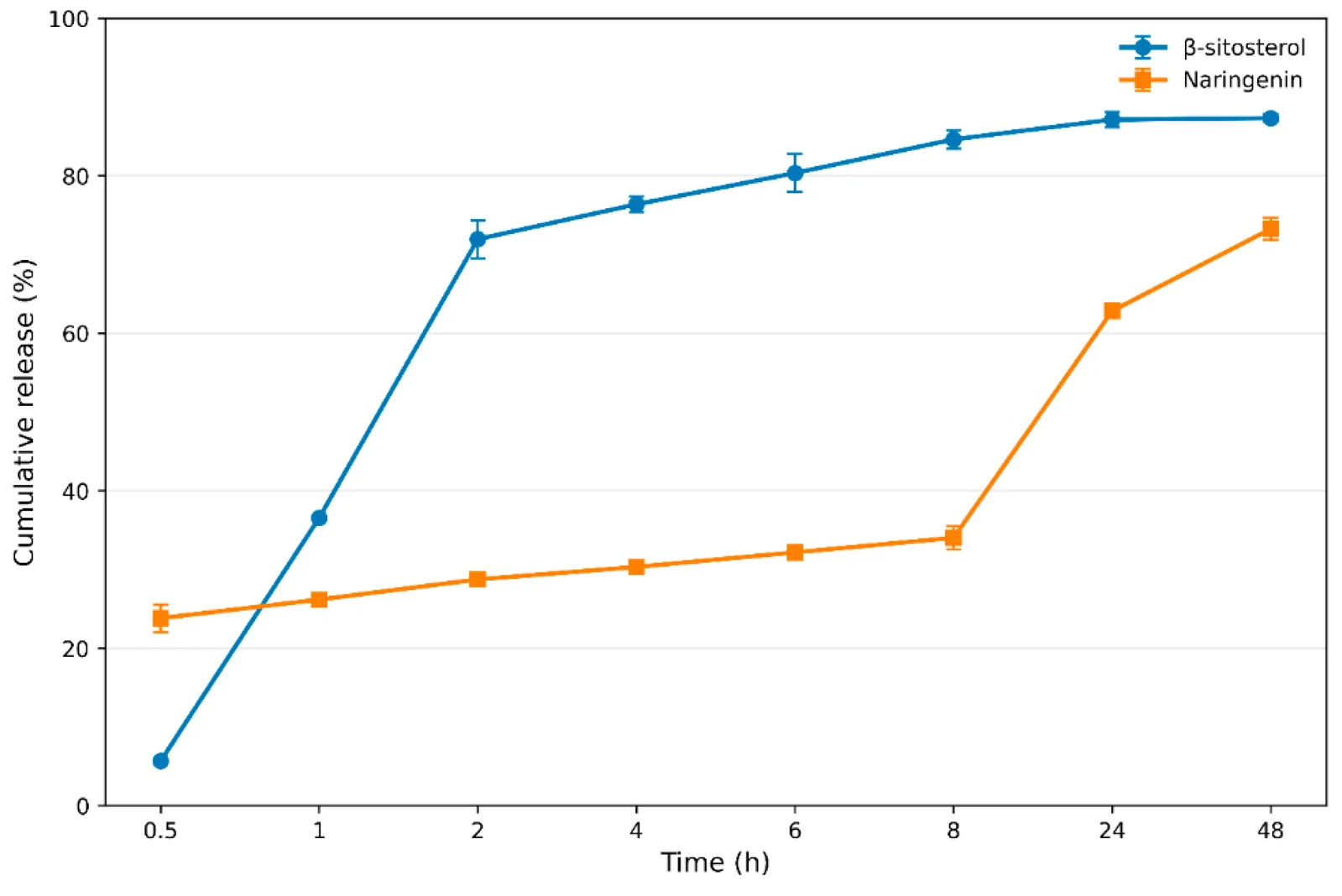
Experimental and augmented cumulative release profiles of β-sitosterol and naringenin generated using SD-informed and Weibull-based augmentation.

The augmentation procedure generated 1200 release profiles, comprising 600 profiles for each compound. For each compound, 300 profiles were generated using SD-informed sampling and 300 using Weibull-based augmentation. The augmented populations reproduced the overall temporal trajectories of the experimentally observed cumulative release profiles while propagating the experimentally specified variability (Figure 6). All generated values remained within the predefined cumulative-release range of 0–100%.

All augmented release values remained within the physically meaningful range of cumulative release.

#### 2.4.3. Weibull Kinetic Characterization

**Figure 7:**
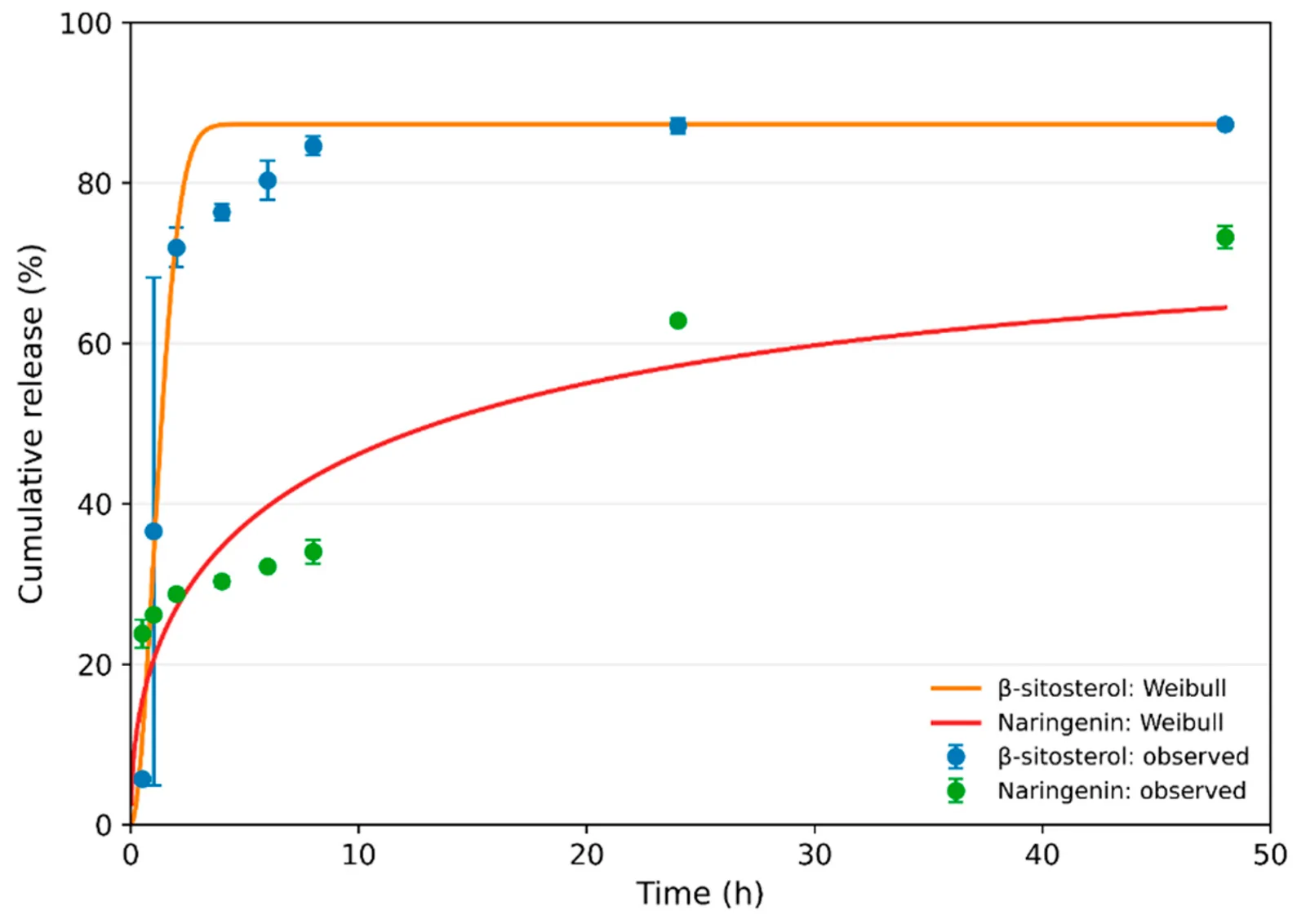
Representative Weibull fits of the cumulative release profiles of β-sitosterol and naringenin.

Representative Weibull fits for the cumulative release profiles of β-sitosterol and naringenin are shown in Figure 7.

The fitted profiles illustrated distinct release behavior between the two compounds, with β-sitosterol showing a more rapid approach toward its estimated release plateau, whereas naringenin exhibited a slower and more gradual release pattern. To quantify these differences across the augmented release populations, the distributions of the Weibull parameters were summarized using the median and interquartile range (Table 1).

β-Sitosterol exhibited a median estimated asymptotic cumulative release (Q∞) of 86.60%, together with a median scale parameter (α) of 1.43 h, a median shape parameter (β) of 3.23, and a median half-release time (t50) of 1.18 h. The corresponding median goodness-of-fit was R^2^ = 0.994.

Naringenin exhibited a median Q∞ of 70.58%, a median α of 8.40 h, a median β of 0.52, and a median t50 of 4.19 h, with a median R^2^ = 0.935.

#### 2.4.4. Comparative Evaluation of Release-Kinetic Models

The fitting performance of the five empirical release models is summarized in Table 2.

For β-sitosterol, the Weibull model yielded the lowest AICc (32.48), the highest Akaike weight (0.753), an R^2^ of 0.966, and an RMSE of 5.10%.

For naringenin, the Higuchi model yielded the lowest AICc (26.38), the highest Akaike weight (0.841), an R^2^ of 0.959, and an RMSE of 3.49%.

The Korsmeyer–Peppas model was fitted only to the initial release phase (≤6 h); therefore, AICc, ΔAICc, and Akaike weights were not calculated for this model.

#### 2.4.5. Early-to-Late Release Prediction

Within the augmented internal test dataset, the direct ridge-regression model showed high predictive agreement for cumulative release at 6, 8, 24, and 48 h using release measurements obtained during the first 4 h (Table 3).

The MAE values ranged from 1.00% to 1.74%, while the RMSE values ranged from 1.17% to 2.09%.

**Figure 8:**
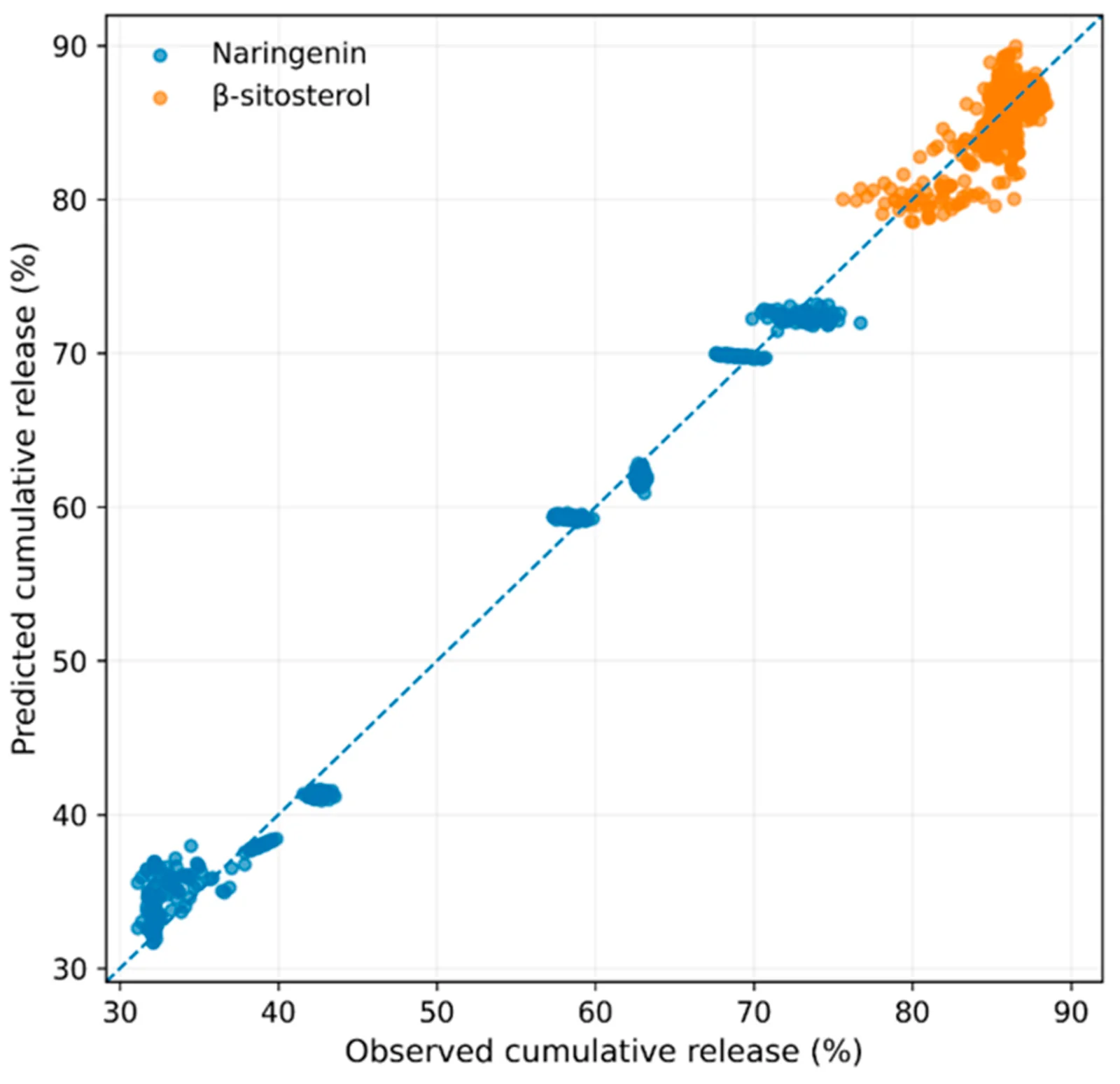
Agreement between observed and ridge-regression-predicted cumulative release values at 6, 8, 24, and 48 h.

The highest predictive performance was observed at 6 h, with an R^2^ of 0.996, an MAE of 1.23%, and an RMSE of 1.57%. Predictions at 8 and 24 h each produced an R^2^ of 0.992. Performance remained high at 48 h, with an R^2^ of 0.976, an MAE of 1.00%, and an RMSE of 1.25%. Predicted values closely followed the corresponding observed values across the evaluated release range (Figure 8).

#### 2.4.6. Weibull-Parameter-Based Release Reconstruction

The ridge-regression model predicted the Weibull parameters with MAE values of 0.910 for Q∞, 0.353 h for α, and 0.431 for β. The corresponding RMSE values were 1.145, 0.544 h, and 0.710, respectively (Table 4).

**Figure 9:**
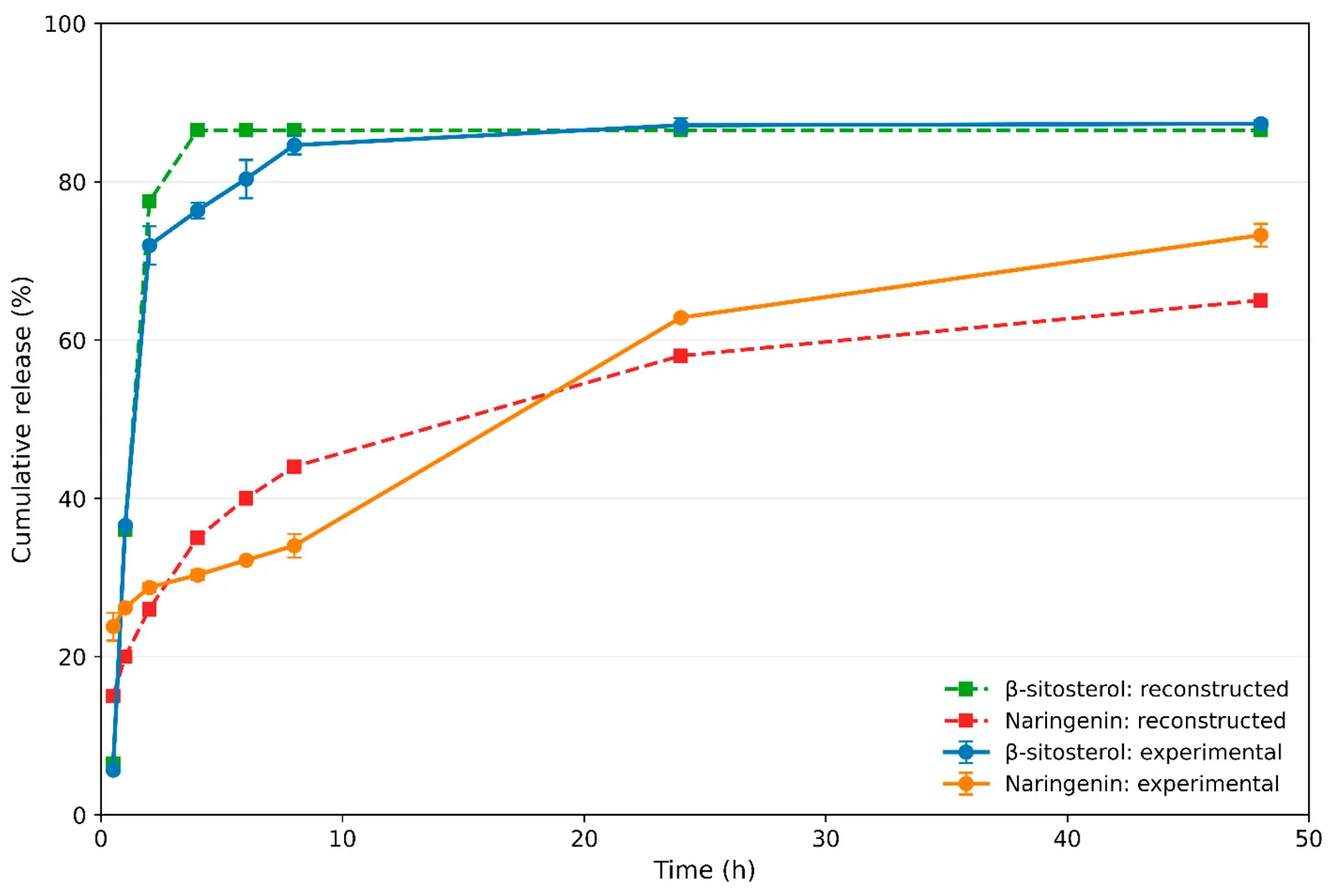
Representative cumulative release profiles reconstructed from ridge-regression-predicted Weibull parameters for β-sitosterol and naringenin.

Release curves reconstructed from the predicted Weibull parameters are presented in Figure 9.

#### 2.4.7. Exploratory Reconstructed-Replicate Holdout Assessment

The additional LOBO analysis evaluated prediction against reconstructed experimental batch trajectories that were held out from model development. Predictive agreement remained high across the experimentally supported later time points (Table 5).

At 6 h, the model yielded an MAE of 4.95%, RMSE of 6.99%, and *R*^2^ of 0.916. At 24 h, the corresponding values were 2.51%, 3.50%, and 0.917, respectively, while at 48 h they were 2.49%, 2.71%, and 0.853. Across all 12 held-out predictions, pooled performance was MAE = 3.32%, RMSE = 4.78%, and *R*^2^ = 0.938.

Reversing the uncertain β-sitosterol 0.5-h batch assignment produced essentially unchanged pooled performance (MAE = 3.32%, RMSE = 4.77%, R^2^ = 0.938), indicating that the overall LOBO result was insensitive to this assignment.

### 2.5. In Vitro Cell Viability Assessment

#### 2.5.1. A549 Lung Adenocarcinoma Cells

β-Sitosterol reduced A549 cell viability in a concentration-dependent manner. The β-sitosterol nanophytosomal formulation produced greater reductions in cell viability than the free compound at concentrations of 26–100 µg/mL, with significance levels ranging from *p* < 0.01 to *p* < 0.0001. No significant differences between the free and nanophytosomal forms were observed at 6 or 200 µg/mL.

Free naringenin produced comparatively limited reductions in A549 cell viability, whereas the nanophytosomal formulation significantly reduced viability at all tested concentrations (*p* < 0.0001). The difference between the free and formulated forms was particularly evident at concentrations of 100 and 200 µg/mL.

**Figure 10:**
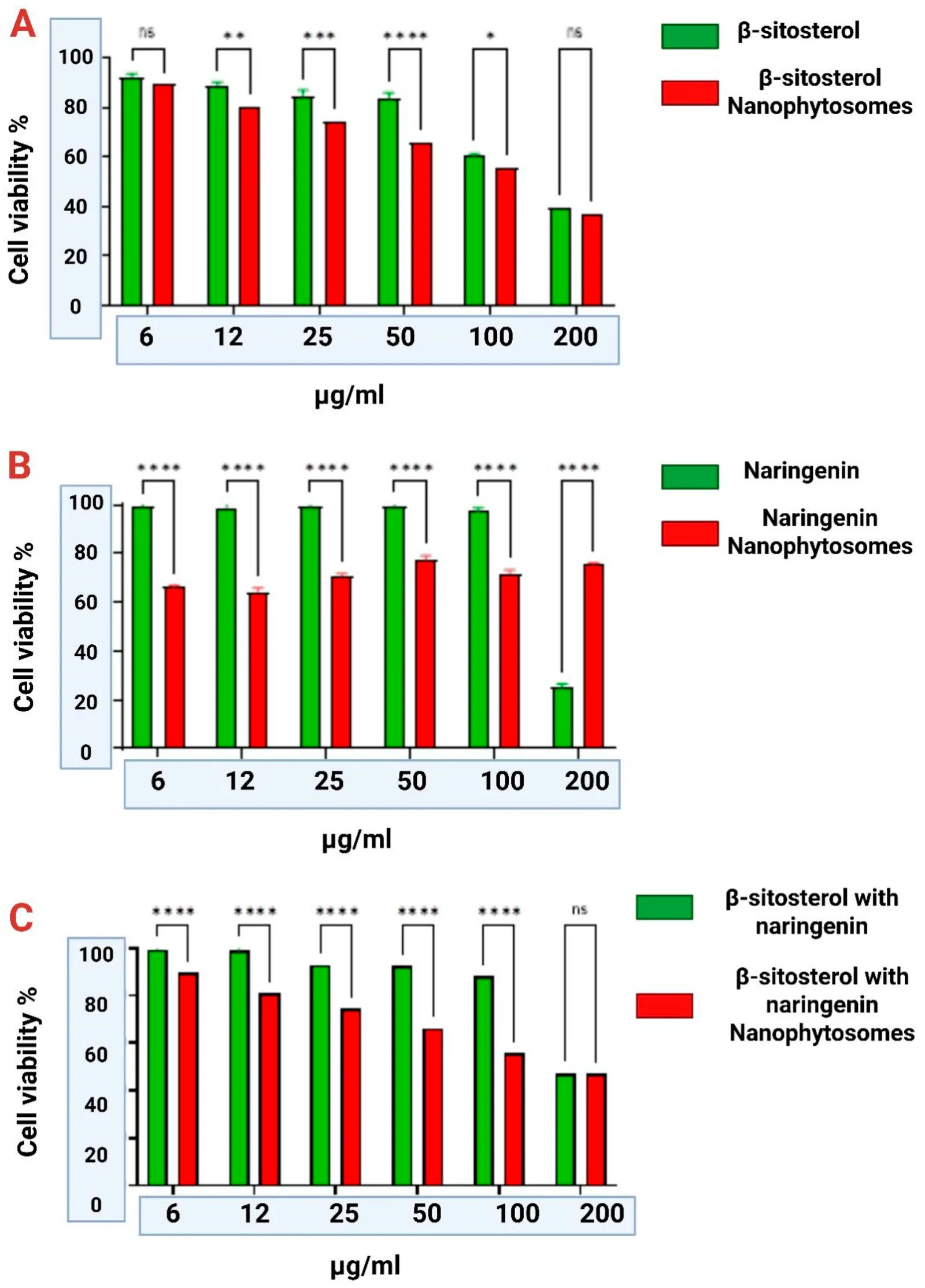
Effects of free and nanophytosome-loaded β-sitosterol and naringenin on A549 lung adenocarcinoma cell viability. (**A**) Free β-sitosterol versus β-sitosterol-loaded nanophytosomes; (**B**) free naringenin versus naringenin-loaded nanophytosomes; (**C**) free β-sitosterol–naringenin combination versus co-loaded nanophytosomes. Cell viability was evaluated using the MTT assay after 72 h of treatment at the indicated concentrations. Data are expressed as mean ± standard deviation (SD; n = 3). Statistical comparisons were performed between free and corresponding nanophytosomal treatments at each concentration. Statistical significance: ns, *p* ≥ 0.05; * *p* < 0.05; ** *p* < 0.01; *** *p* < 0.001; **** *p* < 0.0001.

The free and nanophytosomal combinations of β-sitosterol and naringenin both reduced A549 cell viability. The co-loaded nanophytosomal formulation produced the greatest reduction between 6 and 100 µg/mL, with significant differences relative to the free combination (*p* < 0.0001). No significant difference between the two combination treatments was observed at 200 µg/mL (Figure 10).

#### 2.5.2. HCT116 Colorectal Carcinoma Cells

β-Sitosterol reduced HCT116 cell viability in a concentration-dependent manner. At all evaluated concentrations, the nanophytosomal β-sitosterol formulation produced a greater reduction in cell viability than the free compound (*p* < 0.0001).

Free naringenin and its nanophytosomal formulation produced limited effects at concentrations of 6–60 µg/mL, with no significant differences between the two forms. At 100 and 200 µg/mL, free naringenin produced lower cell viability than the nanophytosomal formulation (*p* < 0.0001).

**Figure 11:**
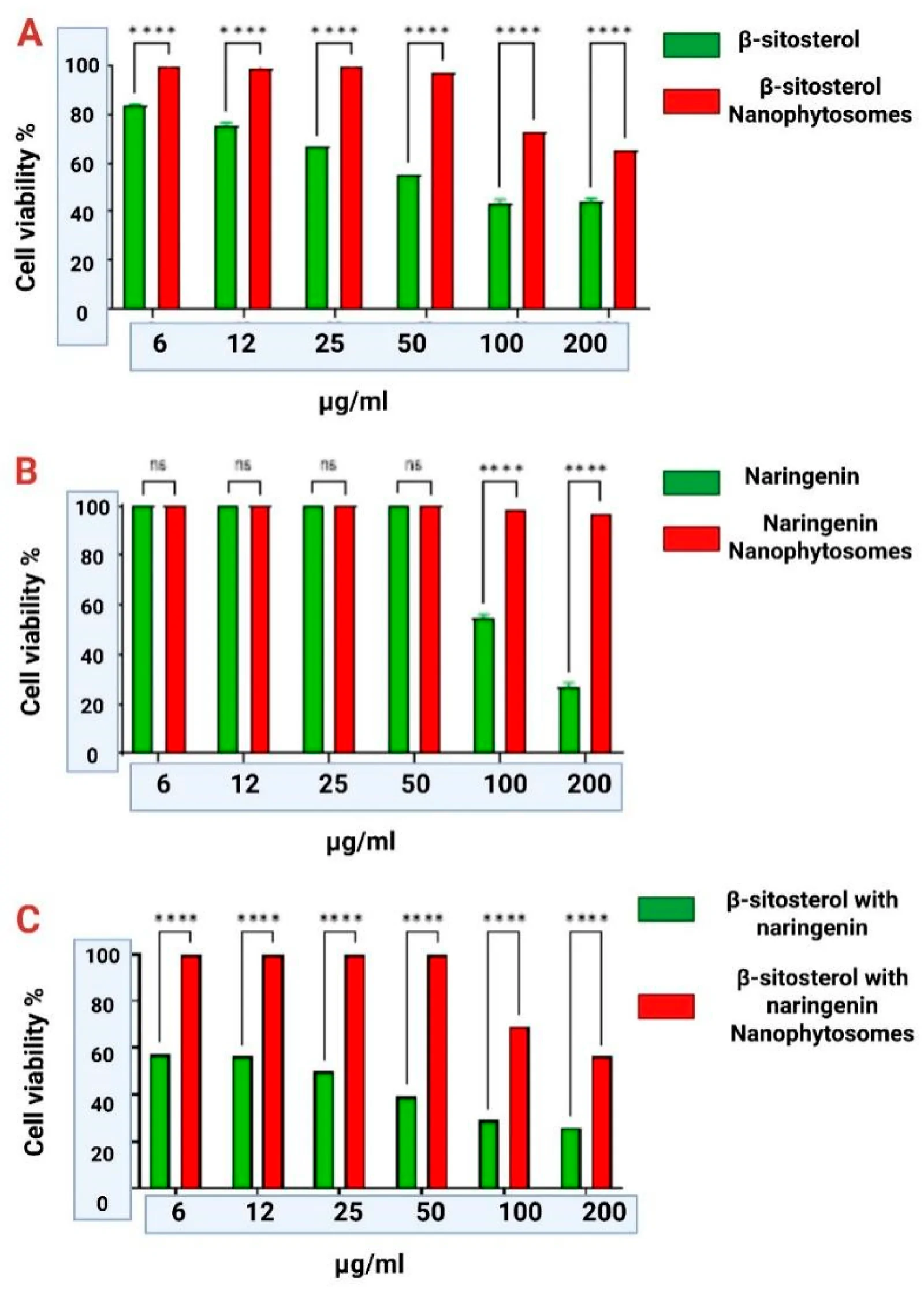
Effects of free and nanophytosome-loaded β-sitosterol and naringenin on HCT116 colorectal carcinoma cell viability. (**A**) Free β-sitosterol versus β-sitosterol-loaded nanophytosomes; (**B**) free naringenin versus naringenin-loaded nanophytosomes; (**C**) free β-sitosterol–naringenin combination versus co-loaded nanophytosomes. Cell viability was evaluated using the MTT assay after 72 h of treatment at the indicated concentrations. Data are expressed as mean ± standard deviation (SD; n = 3). Statistical comparisons were performed between free and corresponding nanophytosomal treatments at each concentration. Four asterisks (****) indicate *p* < 0.0001; ns indicates no statistically significant difference (*p* ≥ 0.05).

The free combination of β-sitosterol and naringenin produced lower HCT116 cell viability than the co-loaded nanophytosomal formulation at all tested concentrations (*p* < 0.0001). The difference between the two treatments increased at the higher concentrations (Figure 11).

#### 2.5.3. HEK293 Cells

At concentrations of 6 and 12 µg/mL, free and nanophytosomal β-sitosterol produced comparable effects on HEK293 cell viability. At concentrations of 26–60 µg/mL, the nanophytosomal formulation produced greater reductions in viability than the free compound, whereas the effects of the two forms were comparable at 100 and 200 µg/mL.

Free naringenin and its nanophytosomal formulation did not significantly differ in their effects on HEK293 cell viability at concentrations of up to 60 µg/mL. At 100 and 200 µg/mL, free naringenin produced lower cell viability than the nanophytosomal formulation (*p* < 0.0001).

**Figure 12:**
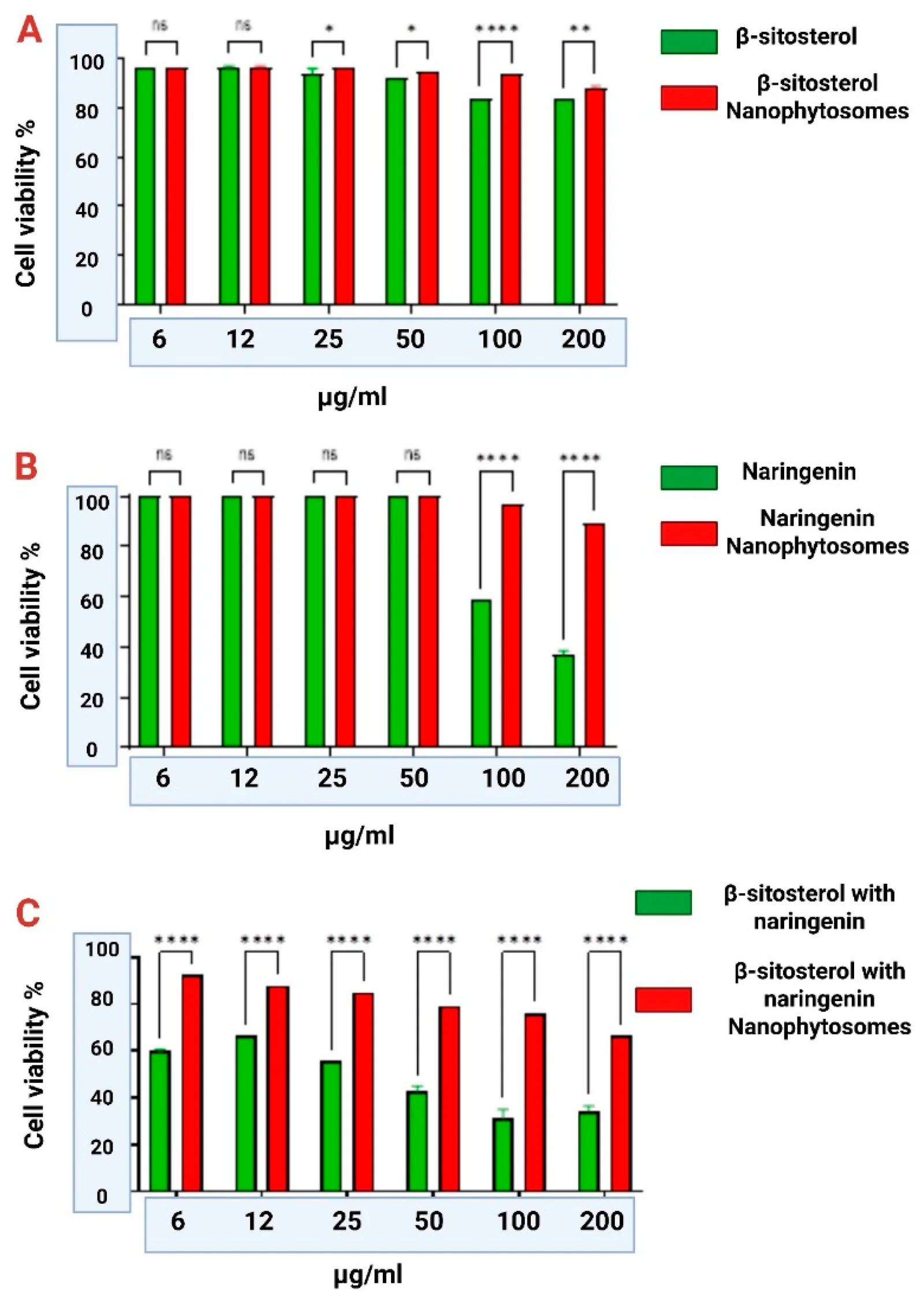
Effects of free and nanophytosome-loaded β-sitosterol and naringenin on HEK293 human embryonic kidney cell viability. (**A**) Free β-sitosterol versus β-sitosterol-loaded nanophytosomes; (**B**) free naringenin versus naringenin-loaded nanophytosomes; (**C**) free β-sitosterol–naringenin combination versus co-loaded nanophytosomes. Cell viability was evaluated using the MTT assay after 72 h of treatment at the indicated concentrations. Data are expressed as mean ± standard deviation (SD; n = 3). Statistical comparisons were performed between free and corresponding nanophytosomal treatments at each concentration. Statistical significance: ns, *p* ≥ 0.05; * *p* < 0.05; ** *p* < 0.01; **** *p* < 0.0001.

The free combination of β-sitosterol and naringenin produced lower HEK293 cell viability than the co-loaded nanophytosomal formulation at all evaluated concentrations (*p* < 0.0001), indicating greater preservation of HEK293 cell viability by the co-loaded formulation under the tested conditions (Figure 12).

### 2.6. Molecular Docking Analysis of β-Sitosterol and Naringenin Against Selected Cancer-Related Targets

#### 2.6.1. Docking Protocol Validation

Redocking reproduced the crystallographic reference poses within the predefined 2.0 Å RMSD criterion for all three targets. Alpelisib redocked into PI3Kα with an RMSD of 0.36 Å, while rofecoxib redocked into COX-2 with an RMSD of 0.82 Å. For MEK1, the reference inhibitor 3BM yielded an all-heavy-atom RMSD of 1.87 Å. Notably, its aromatic core was reproduced with an RMSD of 0.194 Å, whereas the larger deviations were localized primarily to the flexible dihydroxypropoxy substituent. Thus, the principal bound orientation and pharmacophoric core of the reference ligand were retained despite conformational variability in the flexible peripheral chain. Collectively, these redocking results supported the suitability of the three docking protocols for subsequent comparative evaluation of β-sitosterol and naringenin.

#### 2.6.2. Comparative Docking Performance and Pose Placement

Naringenin generated docked poses at all three targets, with Glide scores ranging from −7.58 to −8.40 kcal/mol (Table 6). The most favorable score was obtained at COX-2 (−8.40 kcal/mol), corresponding to 87% of the rofecoxib reference score (−9.65 kcal/mol). Naringenin scored −7.58 kcal/mol at PI3Kα compared with −10.81 kcal/mol for alpelisib and −7.61 kcal/mol at MEK1 compared with −12.28 kcal/mol for 3BM. β-Sitosterol produced scores of −5.45 and −5.85 kcal/mol at PI3Kα and MEK1, respectively, while no stable pose was obtained at COX-2.

The spatial positions of the docked compounds relative to the crystallographic reference ligands were further quantified using centroid displacement (Table 7). Naringenin showed centroid displacements of 1.01 Å at MEK1, 0.72 Å at COX-2, and 2.50 Å at PI3Kα. The corresponding values for β-sitosterol were 2.31 Å at MEK1 and 0.82 Å at PI3Kα; no value was available for COX-2 because no pose was obtained.

**Figure 13:**
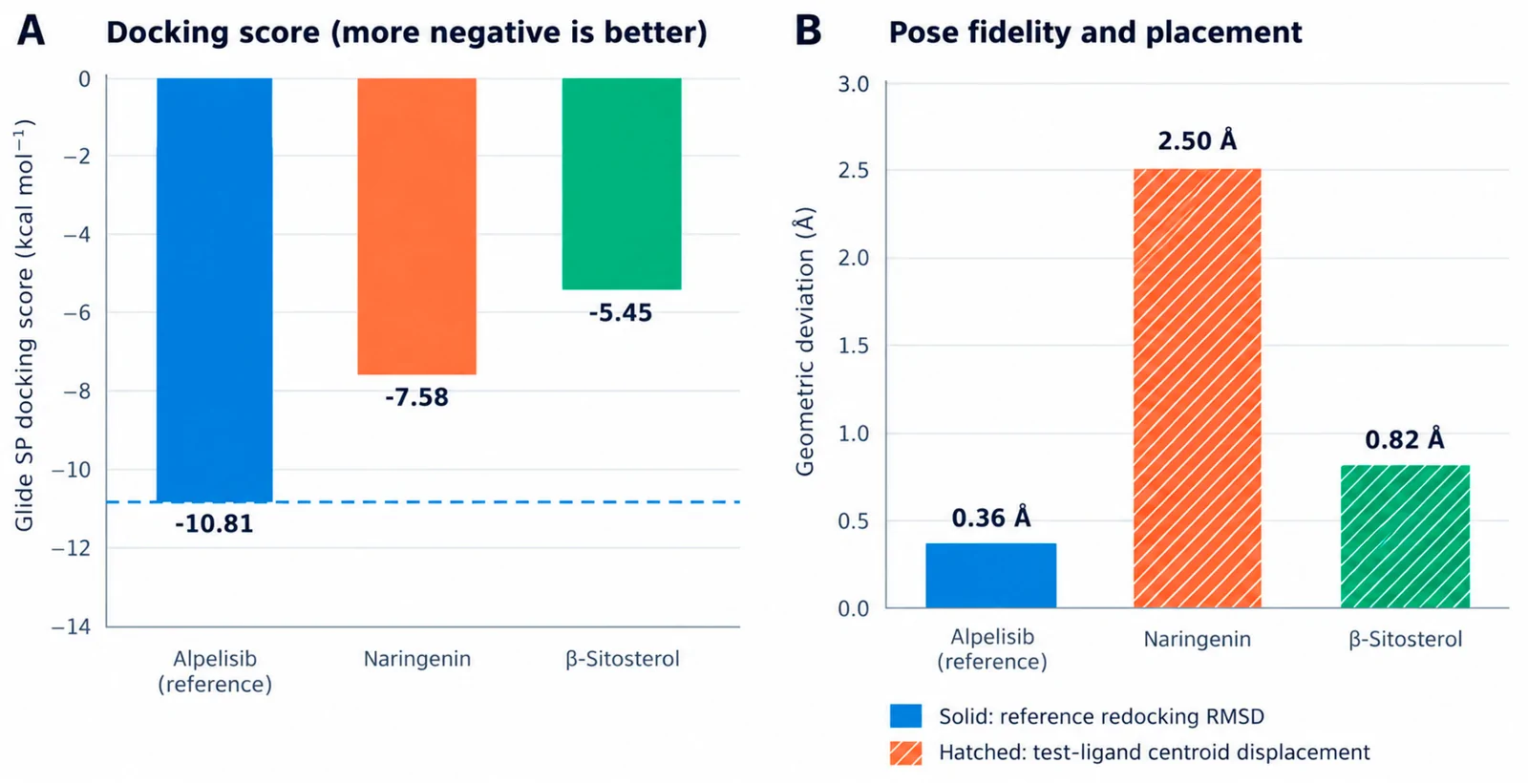
Comparative molecular docking of naringenin and β-sitosterol against PI3Kα (PDB ID: 4JPS) in the context of HCT116 cells. (**A**) Glide standard-precision (SP) docking scores relative to the reference inhibitor alpelisib. The dashed blue line indicates the reference-ligand docking score. (**B**) Redocking root-mean-square deviation (RMSD) of alpelisib from its crystallographic pose (0.36 Å; solid bar) and centroid displacements of naringenin (2.50 Å) and β-sitosterol (0.82 Å) relative to the crystallographic reference ligand (hatched bars). The predefined acceptance criterion of RMSD ≤ 2.0 Å applies exclusively to reference-ligand redocking validation, not to test-compound centroid displacement.

At PI3Kα, naringenin and β-sitosterol produced docking scores of −7.58 and −5.45 kcal/mol, respectively, compared with −10.81 kcal/mol for alpelisib (Figure 13). Their centroid displacements from the crystallographic reference ligand were 2.50 and 0.82 Å, respectively, while alpelisib redocking yielded an RMSD of 0.36 Å.

**Figure 14:**
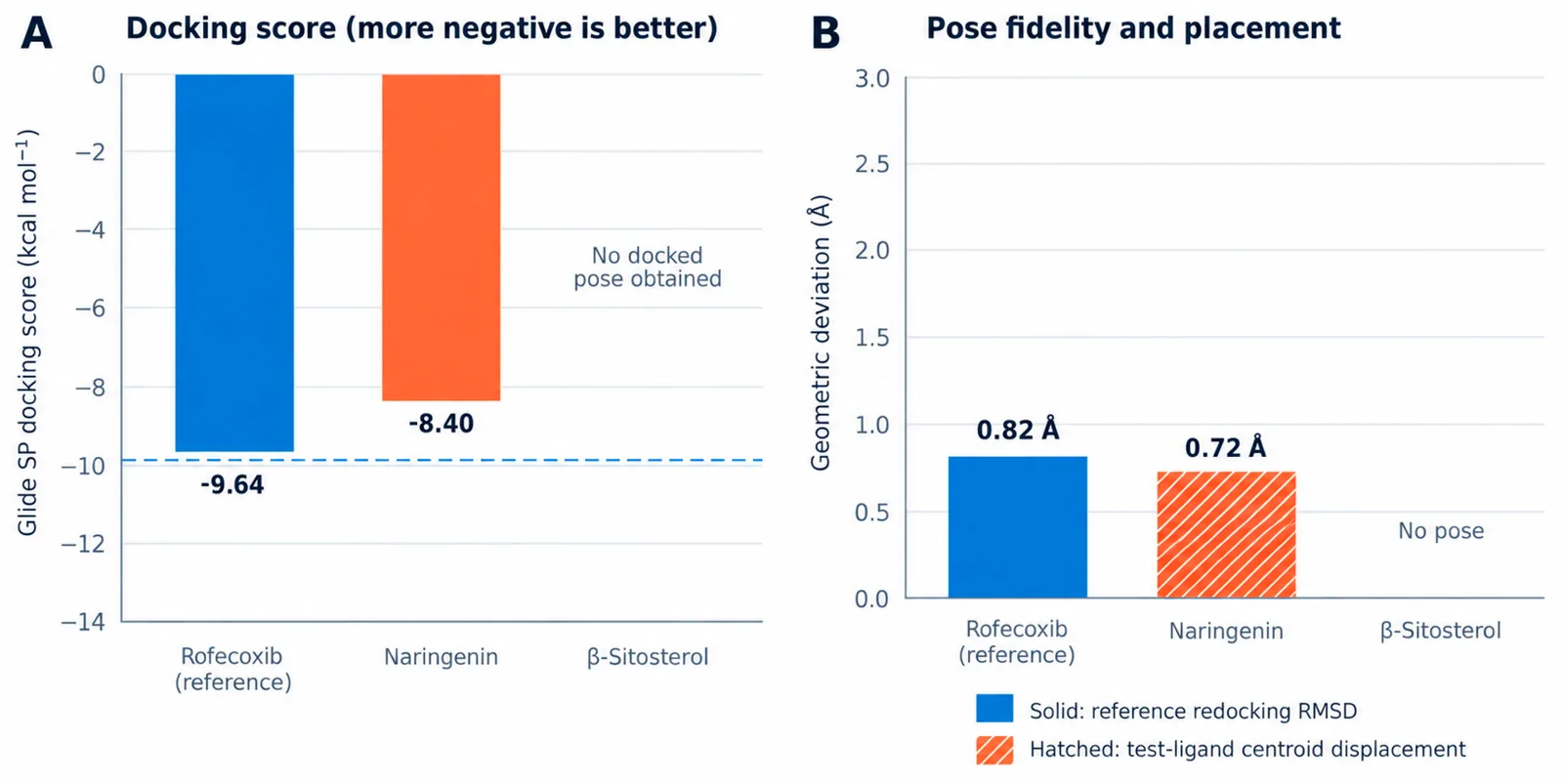
Comparative molecular docking of naringenin and β-sitosterol against COX-2 (PDB ID: 5KIR) in the context of A549 cells. (**A**) Glide SP docking scores relative to the reference inhibitor rofecoxib, with the dashed blue line indicating its docking score. (**B**) Redocking RMSD of rofecoxib (0.82 Å; solid bar) and centroid displacement of naringenin (0.72 Å; hatched bar) relative to the crystallographic reference ligand. β-Sitosterol yielded no docked pose under the evaluated conditions, including expanded grid dimensions and conformational sampling. The RMSD ≤ 2.0 Å validation criterion applies only to reference-ligand redocking.

At COX-2, naringenin produced a docking score of −8.40 kcal/mol compared with −9.65 kcal/mol for rofecoxib, corresponding to 87% of the reference score (Figure 14). The naringenin pose showed a centroid displacement of 0.72 Å, whereas the redocked rofecoxib pose had an RMSD of 0.82 Å. β-Sitosterol did not yield a stable docked pose at this target.

**Figure 15:**
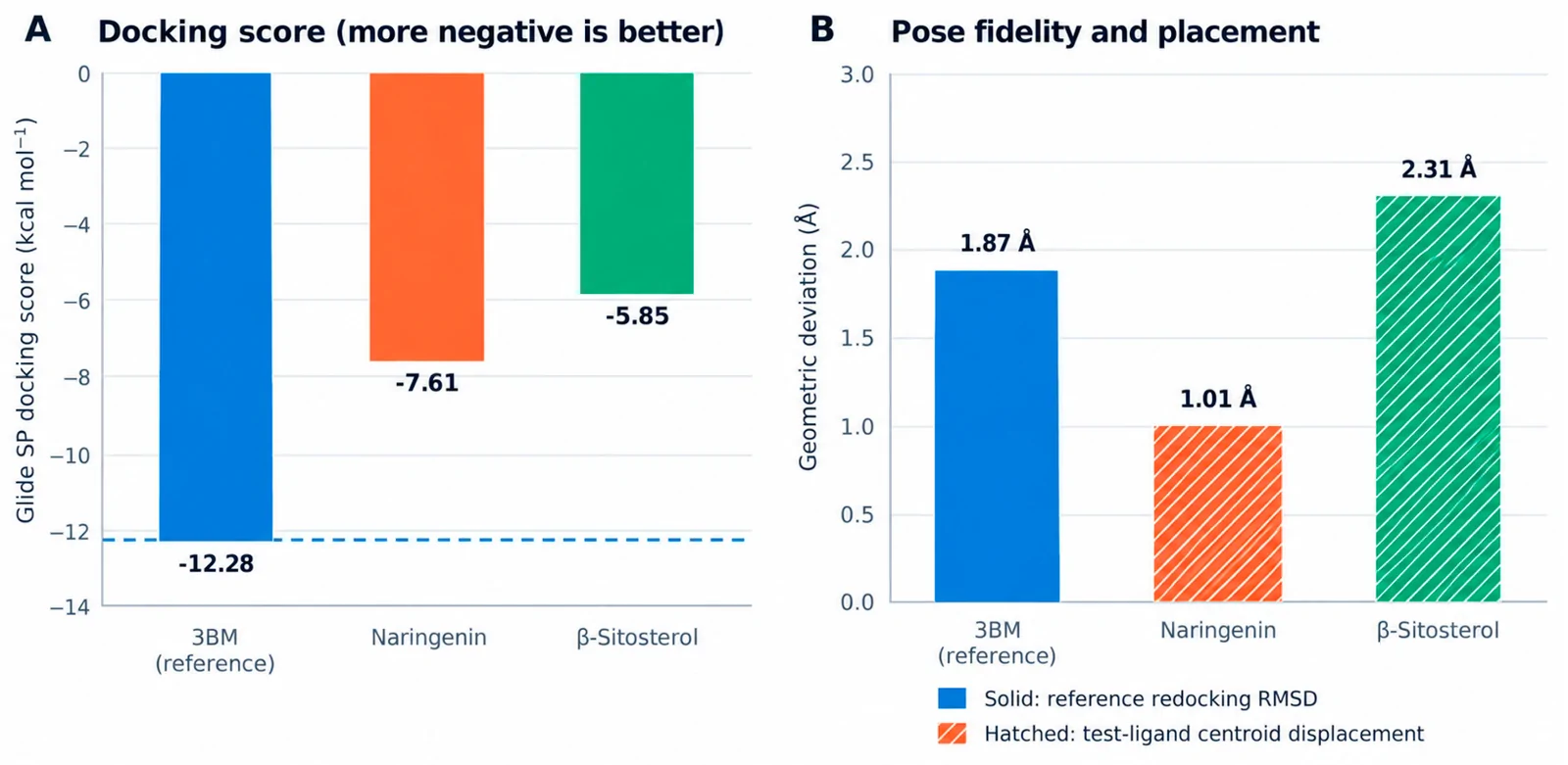
Comparative molecular docking of naringenin and β-sitosterol against MEK1 (PDB ID: 3EQC) in the context of A549 cells. (**A**) Glide SP docking scores relative to the crystallographic allosteric inhibitor 3BM, with the dashed blue line indicating the reference-ligand docking score. (**B**) All-heavy-atom redocking RMSD of 3BM (1.87 Å; solid bar) and centroid displacements of naringenin (1.01 Å) and β-sitosterol (2.31 Å) relative to the crystallographic reference ligand (hatched bars). The predefined RMSD ≤ 2.0 Å criterion was used exclusively to assess reference-ligand pose reproduction and does not represent an acceptance threshold for test-compound centroid displacement.

At MEK1, naringenin and β-sitosterol showed docking scores of −7.61 and −5.85 kcal/mol, respectively, compared with −12.28 kcal/mol for 3BM (Figure 15). These values corresponded to 62% and 48% of the reference score, respectively. Their centroid displacements were 1.01 Å for naringenin and 2.31 Å for β-sitosterol, while the reference ligand redocked with an all-heavy-atom RMSD of 1.87 Å.

#### 2.6.3. Binding-Site Interaction Analysis

##### PI3Kα Binding Interactions

The best-scoring naringenin pose at PI3Kα formed hydrogen-bond interactions with the Val851 backbone at 2.03 and 1.97 Å and with Asp933 at 1.96 Å (Table 8). Hydrophobic contacts involved Ile800, Ile848, Trp780, Met922, and Ile932. The pose contained 223 good contacts, four bad contacts, and no severe contacts. β-Sitosterol formed a single hydrogen bond with Asp933 at 1.70 Å and hydrophobic contacts with Met922, Trp780, Val850, Ile800, Ile932, Ile848, Met772, and Arg852; 424 good, 42 bad, and four severe contacts were recorded. The reference ligand alpelisib interacted with Gln859, Val851, Ser854, and Trp780 and showed 357 good, six bad, and one severe contact.

**Figure 16:**
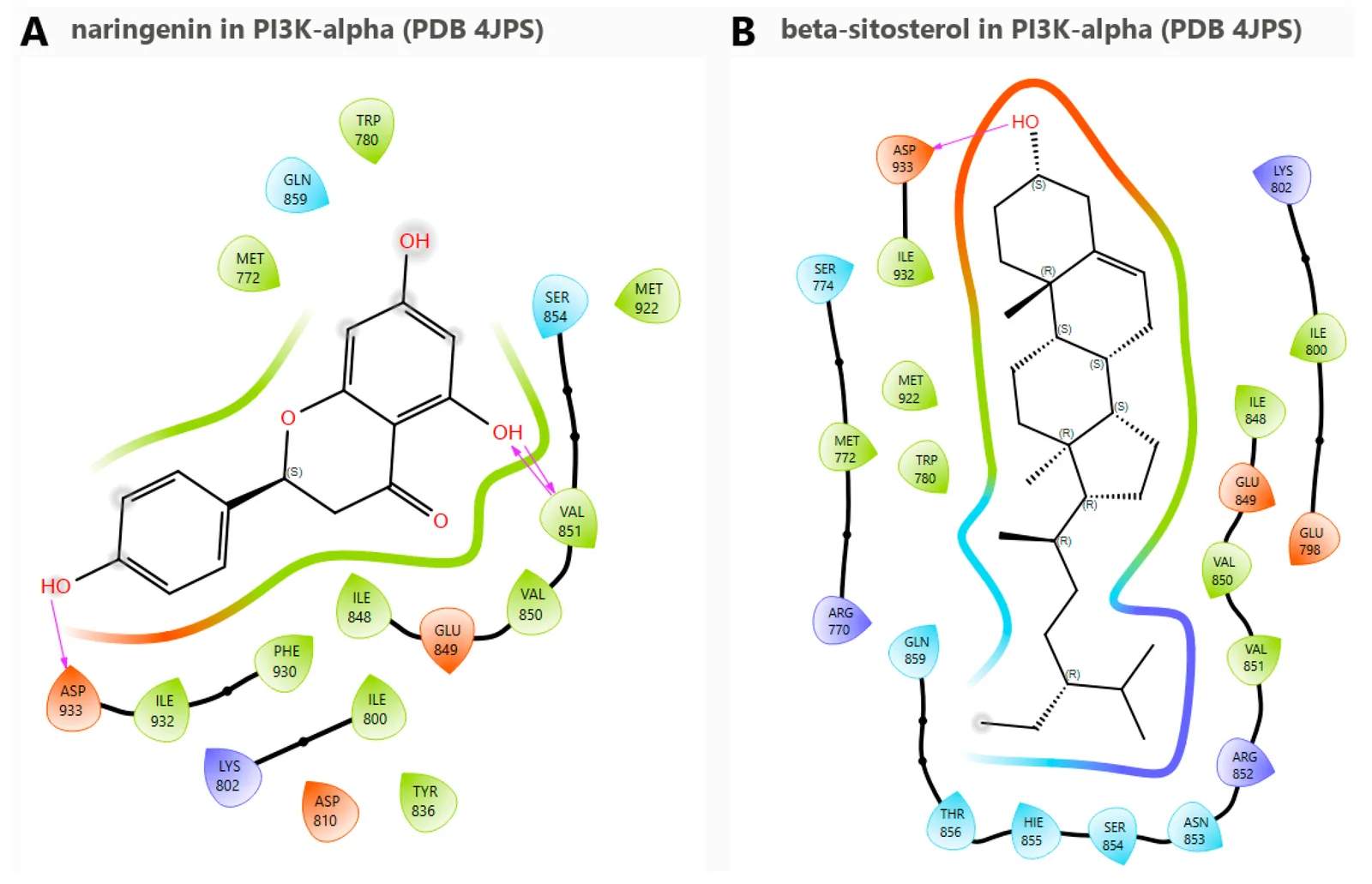
Ligand interaction diagrams at PI3Kα (PDB 4JPS). (**A**) Naringenin, showing interactions with Val851 and Asp933; (**B**) β-sitosterol, showing an interaction with Asp933 and surrounding hydrophobic contacts.

The corresponding interaction diagrams are presented in Figure 16. Naringenin displayed the Val851 and Asp933 interactions identified in the contact analysis, whereas β-sitosterol showed the Asp933 interaction together with predominantly hydrophobic contacts.

##### COX-2 Binding Interactions

At COX-2, naringenin formed hydrogen-bond interactions with Ser530 (1.61 Å), Gln192 (1.98 Å), His90 (2.23 Å), and Tyr355 (Table 9). Hydrophobic contacts involved Val523, Phe518, Leu352, Trp387, and Val349. The naringenin pose contained 285 good, 10 bad, and one severe contact. The reference ligand rofecoxib formed an interaction with Arg513 at 1.78 Å and an aromatic interaction with Met522, together with hydrophobic contacts involving Val349, Ala527, Leu352, Trp387, Val523, Phe518, and Ala516. β-Sitosterol produced no docked pose at this target.

**Figure 17:**
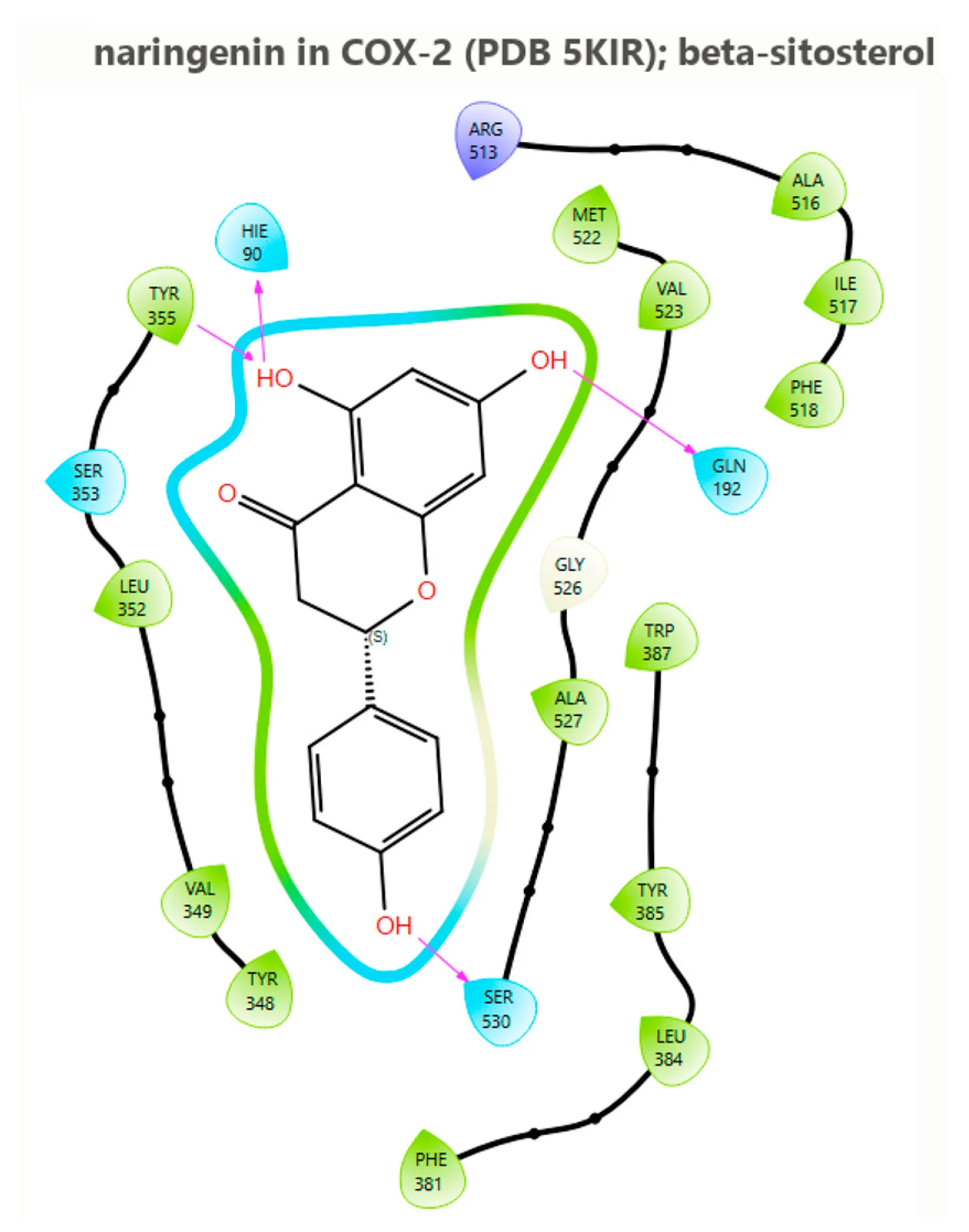
Ligand interaction diagram of naringenin at COX-2 (PDB 5KIR), showing hydrogen-bond interactions with Tyr355, His90, Gln192, and Ser530. β-Sitosterol yielded no docked pose.

The naringenin interaction pattern within the COX-2 binding site is shown in Figure 17, including its contacts with Tyr355, His90, Gln192, and Ser530. No corresponding interaction diagram was generated for β-sitosterol because no stable pose was obtained.

Repeating the β-sitosterol docking procedure using an enlarged inner grid box (15 Å), outer box (40 Å), and expanded sampling again yielded no pose.

##### MEK1 Binding Interactions

At MEK1, naringenin formed interactions with ATP-γ-S at 1.35 and 2.59 Å, while the interaction diagram also showed a hydrogen-bond interaction with Ser212 and a cation–π interaction with Lys97 (Table 10). The naringenin pose contained 270 good, 13 bad, and one severe contact. β-Sitosterol showed a hydrogen-bond interaction between its 3-hydroxyl group and Arg234 in the interaction diagram and exhibited 500 good, 38 bad, and two severe contacts. The reference inhibitor 3BM interacted with Lys97, Asp190, ATP-γ-S, and Phe209 and showed 332 good, 10 bad, and two severe contacts.

**Figure 18:**
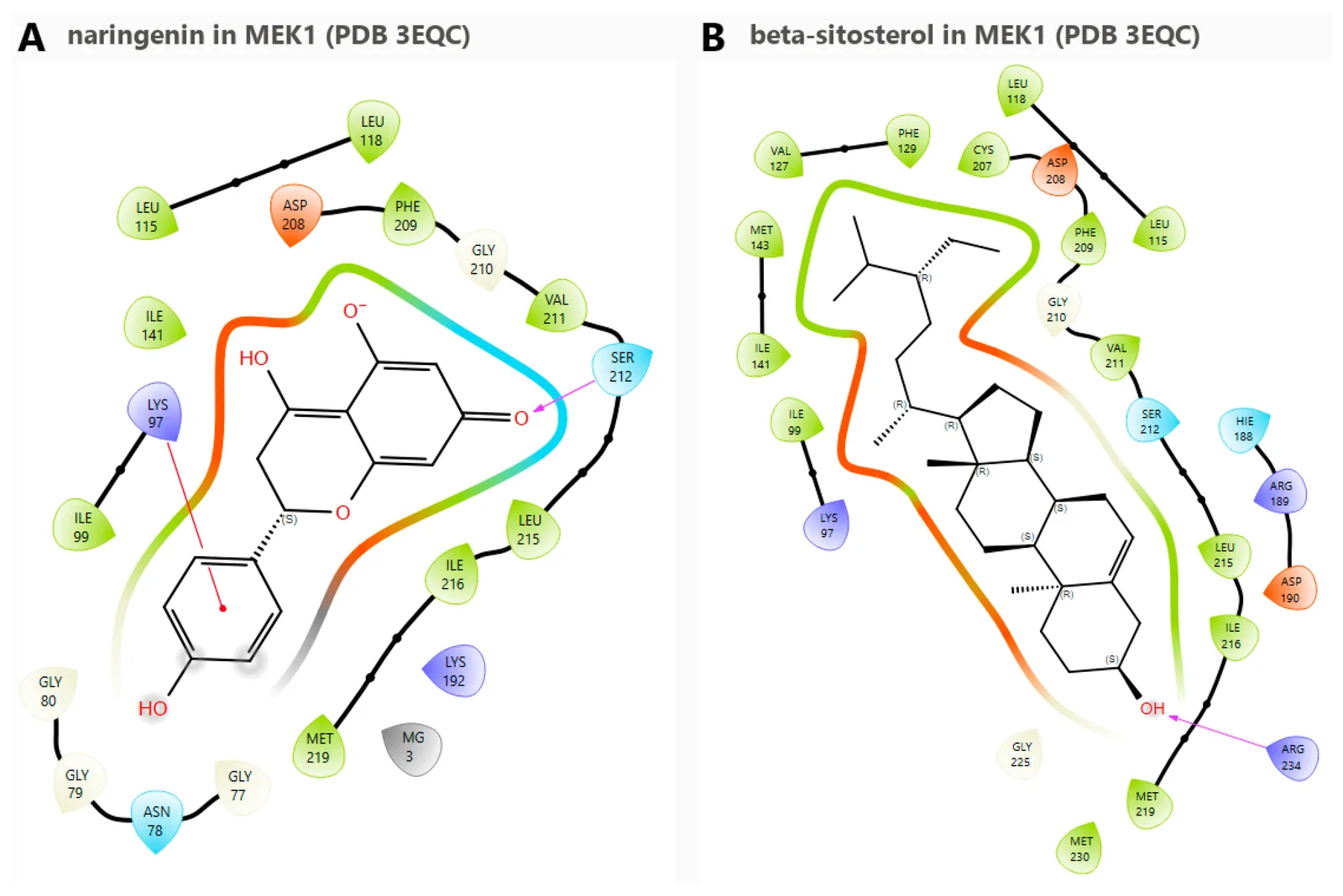
Ligand interaction diagrams at MEK1 (PDB 3EQC). (**A**) Naringenin, showing interactions with ATP-γ-S, Ser212, and Lys97; (**B**) β-sitosterol, showing the interaction with Arg234 and surrounding hydrophobic contacts.

Representative interaction diagrams are shown in Figure 18. Naringenin displayed interactions with ATP-γ-S, Ser212, and Lys97, whereas β-sitosterol showed an interaction with Arg234 together with predominantly nonpolar contacts.

#### 2.6.4. Cross-Target Comparison of Ligand–Protein Interactions

Across the three targets, naringenin generated docked poses at PI3Kα, COX-2, and MEK1, whereas β-sitosterol generated poses at PI3Kα and MEK1 but not at COX-2. Naringenin showed 4, 10, and 13 bad contacts at PI3Kα, COX-2, and MEK1, respectively, with 0, 1, and 1 severe contacts. In comparison, β-sitosterol showed 42 bad and four severe contacts at PI3Kα and 38 bad and two severe contacts at MEK1.

## 3. Discussion

### 3.1. Co-Loading Reshapes Encapsulation and Physicochemical Properties

The principal formulation finding was the opposing effect of co-loading on encapsulation efficiency. Relative to the corresponding single-loaded formulations, β-sitosterol encapsulation increased substantially in the co-loaded formulation, whereas naringenin encapsulation decreased. These opposing trends indicate that the encapsulation behavior of the co-loaded system differs from that of the individual formulations but do not establish the underlying molecular mechanism. Naringenin has previously shown high encapsulation in phospholipid-based nanosystems [22], while interactions between flavonoids and phosphatidylcholine can modify bilayer organization and lipid packing [23]. Differences in compound–phospholipid interactions, competitive incorporation, or formulation-dependent solubilization and retention could plausibly contribute to the observed encapsulation changes. However, the present measurements do not directly demonstrate reciprocal redistribution or localization within specific regions of the phospholipid bilayer; these possibilities therefore remain hypothetical and require dedicated structural or spectroscopic investigation.

Co-loading was also associated with marked changes in particle size and size distribution. Naringenin-loaded nanophytosomes exhibited the largest mean diameter, β-sitosterol-loaded nanophytosomes showed an intermediate size, and the co-loaded formulation produced the smallest particles. These differences may reflect compound-dependent effects on vesicle assembly, potentially related to molecular polarity, steric structure, or interactions with the phospholipid components. Nevertheless, the present measurements do not directly establish the molecular arrangement of either compound within the bilayer, and mechanistic interpretations of the size changes should therefore remain tentative.

Importantly, the reduction in mean particle size after co-loading was not accompanied by increased population homogeneity. Naringenin-loaded nanophytosomes exhibited the lowest PDI (0.235), whereas β-sitosterol-loaded and co-loaded formulations showed broader distributions, with the co-loaded formulation displaying the highest PDI. Thus, mean particle size and dispersity describe distinct formulation properties: the co-loaded system produced the smallest vesicles but not the narrowest size distribution. This distinction is particularly important because the small standard deviation of the mean particle diameter should not be interpreted as evidence of a more homogeneous vesicle population.

All three formulations exhibited moderately negative zeta potentials of approximately −14 to −17 mV, with no significant differences among groups. Accordingly, loading β-sitosterol, naringenin, or both altered particle size and dispersity without producing a statistically detectable change in surface charge. The relatively modest magnitude of the measured zeta potentials also indicates that electrostatic repulsion alone is unlikely to account for the colloidal behavior of these formulations. Because zeta potential is influenced by phospholipid composition, dispersion medium, ionic strength, and interfacial organization, additional stability studies would be required before inferring long-term colloidal stability from these measurements alone.

SEM analysis provided complementary morphological characterization of the lyophilized nanophytosomal formulations. However, because the samples were examined following sucrose-assisted lyophilization and platinum sputter coating, their observed morphology may not fully represent the native vesicular architecture in aqueous dispersion. Accordingly, the SEM findings should be interpreted alongside the DLS measurements, recognizing that dehydration and the presence of a lyoprotectant may influence apparent particle morphology and aggregation. These observations do not independently establish the molecular organization of the encapsulated compounds within the phospholipid bilayer.

### 3.2. Compound-Specific Release Kinetics and Predictive Modeling

The formulation-dependent behavior extended to drug release. β-Sitosterol exhibited limited initial release followed by a more pronounced increase, whereas naringenin showed greater early release followed by a progressively slower phase. The corresponding Weibull parameters further distinguished the temporal profiles, with markedly different shape and scale parameters for the two compounds. Consequently, co-loading produced an evolving rather than fixed β-sitosterol-to-naringenin release ratio. Such asynchronous release is relevant to dual-compound systems because the relative extracellular exposure to the two compounds changes with time rather than remaining proportional to their initially encapsulated amounts. Although Tween 80 supplementation and periodic medium replenishment were employed to promote sink conditions, the equilibrium solubility of the compounds in the selected medium was not independently determined. Consequently, potential contributions from solubility limitations or dialysis-membrane transport resistance to the apparent release plateau cannot be excluded. Furthermore, the computationally augmented profiles presented in Figure 6 represent mathematical reconstructions informed by the experimental measurements in Figure 5 rather than independent evidence of specific release mechanisms.

Comparative kinetic modeling provided complementary mathematical descriptions of these profiles. Weibull provided the strongest full-profile fit for β-sitosterol, while the first-order model retained secondary statistical support. For naringenin, the Higuchi model provided the strongest full-profile description, whereas the Korsmeyer–Peppas model closely described the restricted early-release interval. These findings should be interpreted primarily as empirical kinetic descriptions rather than definitive evidence of specific molecular release mechanisms, because model agreement alone cannot establish the underlying transport process. Evaluating complete and early-phase profiles nevertheless revealed compound-specific kinetic behavior that would have been obscured by relying on R^2^ or a single release model alone [12,13].

The machine learning analysis extended this characterization by testing whether later release could be estimated from early measurements. Within the augmented internal test data, ridge regression showed high predictive agreement across the 6–48 h horizons. Performance decreased, as expected, when evaluation was extended to the exploratory leave-one-batch-out analysis, although pooled agreement remained high across the experimentally supported 6-, 24-, and 48-h endpoints (R^2^ = 0.938; MAE = 3.32%; RMSE = 4.78%). The insensitivity of the pooled result to reversal of the uncertain β-sitosterol 0.5-h batch assignment provided an additional robustness check.

Weibull-parameter reconstruction further reproduced compound-dependent differences in release behavior, with stronger reconstruction for β-sitosterol than for naringenin. This agrees with the conventional model comparison, in which Weibull was the preferred full-profile representation for β-sitosterol but not for naringenin. Importantly, however, the augmented internal test and reconstructed-batch LOBO analyses are not equivalent to prospective external validation. Their value is therefore in demonstrating temporal predictability within the present experimental framework and providing a basis for subsequent validation using independently prepared batches and previously unseen formulations.

### 3.3. Cell-Dependent Effects of Nanophytosomal Formulation

The biological findings demonstrated that formulation effects were strongly dependent on the cellular model. In A549 cells, nanophytosomal formulation increased the viability-reducing activity of both individual compounds, while the co-loaded formulation produced the largest reduction relative to the free combination across several submaximal concentrations. Previous studies have likewise reported activity of nanoformulated naringenin and of β-sitosterol and naringenin in A549 cells [15,18,19]. The present findings therefore indicate enhanced activity of the co-loaded formulation under the tested A549 conditions. They should not, however, be interpreted as pharmacological synergy because no formal additive-interaction model was applied.

The response in HCT116 cells differed substantially. β-Sitosterol retained an advantage after nanophytosomal incorporation, whereas naringenin showed no consistent formulation-associated improvement and the free β-sitosterol–naringenin combination produced greater viability reduction than the co-loaded formulation. Given the reported activity of both compounds against HCT116 cells [17,20], these results demonstrate that nanophytosomal incorporation does not uniformly enhance biological activity across cancer-cell models. Differences in release kinetics, cellular uptake, intracellular disposition, or compound availability could contribute to this pattern, but these mechanisms were not directly measured in the present study.

The HEK293 findings provided an additional comparison of formulation-dependent cellular effects. The co-loaded formulation preserved higher HEK293 viability than the corresponding free combination across the evaluated concentrations, while the behavior of the individual compounds remained concentration dependent. Together with the A549 and HCT116 findings, these results demonstrate a cell-dependent rather than universally enhanced effect of co-loading. The most favorable profile was observed in A549 cells, where increased viability reduction relative to the free combination coincided with comparatively greater preservation of HEK293 viability. HEK293 cells, however, should not be considered a substitute for comprehensive normal-tissue safety assessment, and these findings require confirmation in additional nonmalignant cellular and in vivo models.

### 3.4. Molecular Docking Provides Complementary Structural Context

The docking analysis provided a structural context for the cellular observations but did not establish a direct mechanism of action. Naringenin generated poses at PI3Kα, COX-2, and MEK1, with docking scores ranging from −7.58 to −8.40 kcal/mol. Its most favorable result occurred at COX-2, where its score reached 87% of that obtained for the rofecoxib reference ligand. At PI3Kα, naringenin formed interactions with the Val851 hinge region and Asp933, while the COX-2 pose involved Tyr355, His90, Gln192, and Ser530. At MEK1, the predicted interaction pattern was less extensive and included contacts involving ATP-γ-S, Ser212, and Lys97.

β-Sitosterol showed a distinctly different docking profile. It generated less favorable scores at PI3Kα and MEK1 and exhibited substantially larger numbers of unfavorable contacts, while no stable pose was obtained at COX-2 even after expanded sampling. These findings indicate that, among the two compounds, naringenin showed the more consistent predicted compatibility with the selected binding sites. Nevertheless, the reference ligands generally retained considerably more favorable scores, and docking scores and poses alone cannot demonstrate target engagement, inhibition, or pathway modulation in cells [24].

### 3.5. Integrated Interpretation, Limitations, and Future Directions

Taken together, the study shows that co-loading β-sitosterol and naringenin modifies several interconnected properties of the nanophytosomal system. The formulation exhibited reciprocal changes in encapsulation efficiency, a reduction in mean particle size accompanied by broader dispersity, compound-specific release behavior, and a time-dependent delivery ratio. Kinetic modeling characterized these distinct release patterns, while machine learning analysis demonstrated that later release could be estimated from early observations within the present experimental framework.

These physicochemical differences were accompanied by cell-dependent biological responses rather than a uniform enhancement of activity. The co-loaded formulation showed its most favorable comparative profile in A549 cells, whereas the free combination remained more active in HCT116 cells. Docking further identified naringenin as the compound with the more consistent predicted interactions across the selected molecular targets, although these computational findings should not be interpreted as evidence of direct target engagement.

Several limitations should frame interpretation of these findings. The molecular organization of the compounds within the phospholipid bilayer was not directly characterized; cellular uptake, intracellular drug concentrations, apoptosis, cell-cycle effects, and pathway-specific responses were not measured; formal combination-index or isobolographic analyses were not performed; and the biological evaluation was restricted to in vitro cellular models. Because the cytotoxicity experiments primarily compared treatment formulations rather than defined-ratio, component-matched dose–effect combinations, the observed responses cannot be conclusively classified as synergistic, additive, or antagonistic.

The predictive framework was developed using augmented profiles derived from a limited experimental dataset. Although this approach enabled systematic exploration of release predictability under experimentally informed variability, it cannot fully capture independent batch-to-batch differences or unmeasured temporal dependencies, potentially yielding optimistic internal performance estimates. Nevertheless, the complementary reconstructed-replicate assessment supported within-system predictive consistency, establishing a proof-of-concept basis for subsequent validation using independently prepared formulations.

Accordingly, future work should incorporate direct structural characterization of the co-loaded carrier, independently prepared formulation batches, prospective validation of the release-prediction framework, cellular uptake and mechanistic assays, formal drug-interaction analysis, and subsequent pharmacokinetic and in vivo evaluation. Such studies would determine whether the formulation-dependent advantages identified here translate beyond the present experimental system.

## 4. Materials and Methods

### 4.1. Materials

β-Sitosterol was obtained from Sigma-Aldrich (Taufkirchen, Germany), and naringenin from Nanochemazone (Leduc, AB, Canada). The phospholipid components and excipients used for nanophytosome preparation included distearoylphosphatidylcholine (DSPC; Sigma, St. Louis, MO, USA), cholesterol (Sigma, Taufkirchen, Germany), and polyethylene glycol (PEG; Perrigo, Fort Wayne, IN, USA). HPLC-grade acetonitrile and ethanol were purchased from Honeywell (Shanghai, China), whereas HPLC-grade chloroform was obtained from Chem-Lab (Zedelgem, Belgium). Phosphate-buffered saline (PBS) was supplied by Servicebio (Wuhan, China), and deionized water was used throughout formulation preparation and analytical procedures. Whatman filter paper was used as required. Three human cell lines were used for the in vitro evaluations: A549 lung adenocarcinoma cells (CCL-185™), HCT116 colorectal carcinoma cells (CCL-247™), and HEK293 embryonic kidney-derived cells (CRL-1573™). The HPLC system, membrane extruder, rotary evaporator, centrifuge, and other specialized instruments are described in the corresponding experimental procedures.

### 4.2. Preparation of β-Sitosterol-, Naringenin-, and Co-Loaded Nanophytosomes

β-Sitosterol-loaded, naringenin-loaded, and co-loaded nanophytosomes were prepared using the ethanol-injection method. The formulations contained phospholipid, cholesterol, and PEGylated phospholipid at a molar ratio of 65:30:5, respectively. β-Sitosterol and naringenin were incorporated at final concentrations of 0.80 and 1.12 mg/mL, respectively; the co-loaded formulation contained both compounds at the same concentrations.

For each formulation, the lipid components and the corresponding active compound(s) were dissolved in ethanol to obtain a homogeneous organic phase. The ethanolic phase was then injected into phosphate-buffered saline (PBS; pH 7.0) at a controlled rate of 2 mL/min under continuous stirring, allowing spontaneous formation of the nanophytosomal vesicles. Residual ethanol was subsequently removed under reduced pressure using a rotary evaporator at 50 °C, 720 mmHg, and 30 rpm.

**Figure 19:**
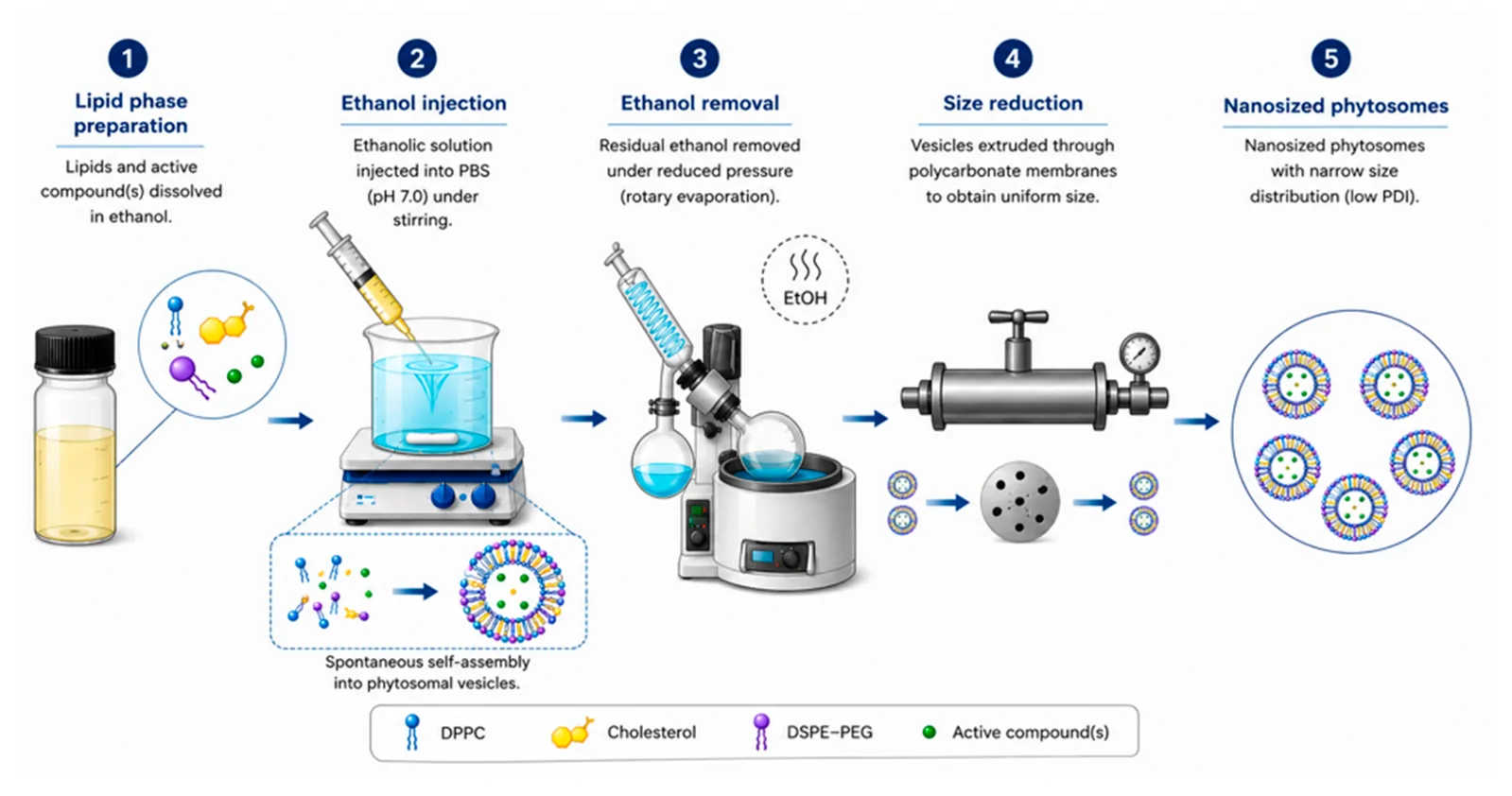
Schematic representation of the preparation of β-sitosterol-loaded, naringenin-loaded, and co-loaded nanophytosomes by ethanol injection, followed by solvent removal and membrane extrusion.

The resulting dispersions were subjected to membrane extrusion to reduce vesicle size and improve formulation consistency. Each formulation was prepared in triplicate and stored under refrigerated conditions at 8 °C until further characterization and analysis. The overall preparation procedure is illustrated in Figure 19.

### 4.3. HPLC Quantification Method of β-Sitosterol and Naringenin

Quantitative determination of β-sitosterol and naringenin was performed using reversed-phase high-performance liquid chromatography (HPLC) equipped with a Fortis C18 analytical column (100 × 4.6 mm; Fortis Technologies Ltd., Neston, Cheshire, UK), maintained at 40 °C. The mobile phase consisted of methanol and water (50:50, *v*/*v*; pH 3.7) and was delivered at a flow rate of 1.0 mL/min. The injection volume was 10 µL, and detection was performed at 210 nm. Under these chromatographic conditions, the retention times were approximately 4.9 min for naringenin and 20.0 min for β-sitosterol [25,26].

Stock solutions of β-sitosterol and naringenin were prepared in ethanol containing a small proportion of chloroform to ensure complete dissolution. Working standard solutions were prepared by serial dilution and used to construct calibration curves by plotting chromatographic peak area against analyte concentration. Quantification of β-sitosterol and naringenin in the experimental samples was subsequently performed using the corresponding calibration relationships. Analyses were conducted in triplicate (n = 3).

### 4.4. Determination of Encapsulation Efficiency

Encapsulation efficiency (EE%) of β-sitosterol and naringenin was determined following the separation of unencapsulated drug from intact nanophytosomes, subsequent vesicle disruption, and HPLC quantification. Briefly, the prepared nanophytosomal suspensions were initially subjected to centrifugation to separate the free-drug fraction from the intact nanophytosomes. The nanophytosome-containing fraction was carefully isolated before further processing. Subsequently, 200 µL of the isolated nanophytosome-containing fraction was mixed with 800 µL of acetonitrile and sonicated for 10 min at 35 °C to ensure complete vesicle disruption and release of the encapsulated compounds. The resulting extract was passed through a 0.45 µm membrane filter prior to HPLC analysis [27].

Encapsulation efficiency was calculated according to Equation (1), and measurements were performed in triplicate (n = 3).

(1) $$\mathrm{EE}\%=\frac{\mathrm{mass}\;\mathrm{of}\;\mathrm{encapsulated}\;\mathrm{drug}}{\mathrm{total}\;\mathrm{drug}\;\mathrm{mass}\;\mathrm{initially}\;\mathrm{added}}\times 100\%$$

### 4.5. Physicochemical Characterization of Nanophytosomes

The physicochemical characteristics of the β-sitosterol-loaded, naringenin-loaded, and co-loaded nanophytosomal formulations were evaluated by measuring particle size, polydispersity index (PDI), and zeta potential using a dynamic light scattering (DLS) Zetasizer (Malvern Panalytical Ltd., Malvern, UK). Prior to analysis, each formulation was diluted 1:20 (*v*/*v*) with deionized water. Measurements were performed in triplicate and expressed as mean ± standard deviation (SD).

#### 4.5.1. Particle Size and Polydispersity Index

The hydrodynamic particle size and PDI of the nanophytosomal formulations were determined by DLS following dilution with deionized water at a sample-to-water ratio of 1:20. Particle size was reported in nanometers (nm), while PDI was recorded as a dimensionless measure of the breadth of the particle-size distribution. All measurements were performed in triplicate.

#### 4.5.2. Zeta Potential

Zeta potential was measured using the same Zetasizer following 1:20 dilution of the formulations with deionized water. Surface-charge values were recorded in millivolts (mV), with measurements performed in triplicate and reported as mean ± SD.

#### 4.5.3. Scanning Electron Microscopy (SEM)

The surface morphology of the nanophytosomal formulations was examined using scanning electron microscopy (SEM). Prior to lyophilization, sucrose was incorporated into the prepared nanophytosomal suspensions as a lyoprotectant to minimize particle aggregation and structural destabilization during freezing and drying. The samples were subsequently pre-frozen in a Lexicon^®^ II ultra-low-temperature freezer (Singapore) and lyophilized using a BÜCHI Lyovapor™ L-200 freeze dryer (BÜCHI Labortechnik AG, Flawil, Switzerland). The resulting lyophilized formulations were collected and prepared for SEM analysis. Before imaging, the dried samples were sputter-coated with an approximately 300 Å-thick platinum layer under an argon atmosphere using an AGAR sputter coater (model AGB7340, Stansted, UK). Morphological characterization was performed using a Phenom XL G2 scanning electron microscope equipped with an AXS energy-dispersive X-ray spectroscopy (EDS) system (Thermo Fisher Scientific, Waltham, MA, USA). SEM images were acquired at a reported vacuum pressure of 6 × 10^−4^ Pa and an accelerating voltage of 15 kV [28].

### 4.6. In Vitro Drug Release

The in vitro release profiles of β-sitosterol and naringenin from the co-loaded nanophytosomal formulation were evaluated using the dialysis-bag diffusion method. Briefly, 1 mL of each nanophytosomal suspension was transferred into a cellulose ester dialysis membrane (molecular weight cut-off: 12,000–14,000 Da), which was subsequently sealed and immersed in 40 mL of phosphate-buffered saline (PBS, pH 7.4) supplemented with 2.5% (*v*/*v*) Tween 80 to enhance the solubility of the released compounds and promote sink conditions. However, equilibrium solubility in the selected medium was not independently determined, precluding quantitative verification of sink conditions throughout the experiment. The system was incubated in a shaking water bath maintained at 37 ± 0.5 °C and 100 rpm. At predetermined intervals (0.5, 1, 2, 4, 6, 24, and 48 h), 1 mL aliquots were withdrawn from the external release medium and immediately replaced with an equivalent volume of prewarmed fresh release medium to maintain a constant total volume. The concentrations of β-sitosterol and naringenin in the collected samples were determined using the HPLC method described previously [29].

The percentage of drug released over time was calculated using Equation (2):

(2) $$\mathrm{Cumulative}\;\mathrm{drug}\;\mathrm{release}\;\left(\%\right)\;=\;\frac{\mathrm{Amount}\;\mathrm{released}}{\mathrm{Total}\;\mathrm{drug}\;\mathrm{loaded}}\times 100$$

### 4.7. Computational Analysis of In Vitro Drug Release

#### 4.7.1. Computational Framework and Dataset Preparation

**Figure 20:**
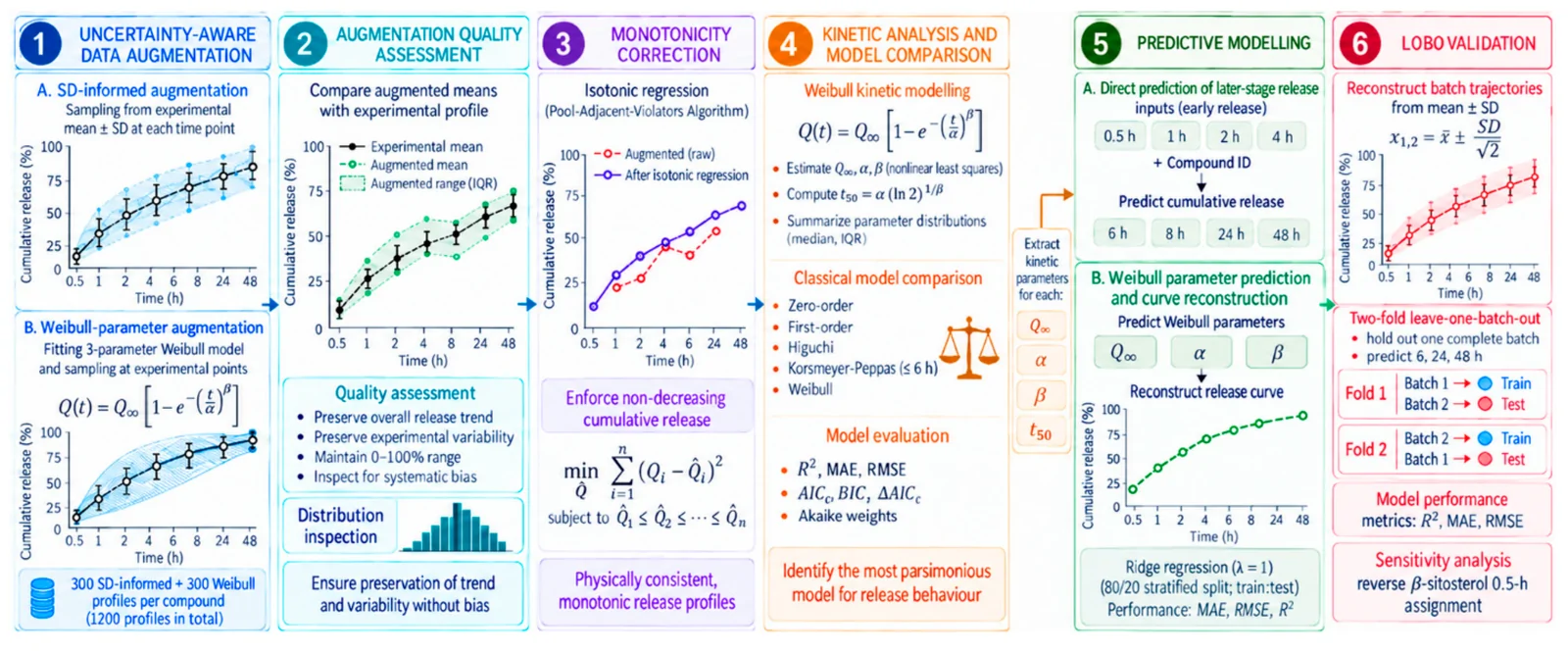
Computational workflow for experimental-data augmentation, kinetic modeling, early-to-late release prediction, internal test evaluation, and exploratory leave-one-batch-out validation.

A computational workflow was developed to complement the experimental release analysis by characterizing compound-specific release kinetics and evaluating whether later cumulative release could be predicted from early measurements. The framework was adapted from our previously reported hybrid predictive-modeling approach for nanoparticle drug-release forecasting [30] and was applied to the present β-sitosterol–naringenin nanophytosomal dataset. The overall workflow comprised experimentally informed data augmentation, kinetic-model fitting, early-to-late release prediction, Weibull-parameter reconstruction, internal test evaluation, and exploratory leave-one-batch-out (LOBO) validation (Figure 20).

The analysis used experimentally determined cumulative release profiles of β-sitosterol and naringenin from the co-loaded nanophytosomal formulation. Release was experimentally measured at 0.5, 1, 2, 4, 6, 24, and 48 h and expressed as mean ± standard deviation (SD). The 8-h release value was estimated using regression rather than obtained through independent experimental measurement and was therefore distinguished from the observed release data.

#### 4.7.2. Summary-Statistic-Based Data Augmentation

To propagate experimentally observed variability and generate datasets of sufficient size for computational modeling, two complementary augmentation strategies were applied.

For SD-informed augmentation, release values at each sampling time were independently sampled from normal distributions parameterized by the experimentally observed mean and SD. Generated values were constrained to the physically meaningful cumulative-release range of 0–100%.

For Weibull-based augmentation, the SD-informed profiles were fitted using the three-parameter Weibull function, in which *Q*_∞_ represents the estimated asymptotic cumulative release, α the scale parameter, and β the shape parameter. Parameters were estimated using bounded nonlinear least-squares optimization, and the resulting fitted curves were sampled at the original experimental time points to generate smooth release trajectories while retaining experimentally informed variability.

For each compound, 300 SD-informed and 300 Weibull-based profiles were generated, yielding 1200 augmented profiles overall. These profiles represent computational realizations derived from the experimentally observed means and standard deviations of duplicate release measurements, rather than additional independent experimental replicates. Augmentation was intended to explore release variability and predictive feasibility within the experimentally informed framework without increasing the effective experimental sample size. Conventional kinetic-model comparisons were performed directly on the observed mean release profiles.

#### 4.7.3. Quality Assessment of Augmented Release Profiles

Before downstream analysis, the augmented release profiles were evaluated against the experimentally observed data. Mean augmented trajectories were compared with the corresponding experimental profiles, and the distributions of generated values were inspected to verify preservation of the observed temporal patterns and experimental variability. Profiles were additionally checked to confirm that cumulative-release values remained within the predefined 0–100% range.

#### 4.7.4. Monotonicity-Constrained Denoising

Because cumulative drug release is expected to be non-decreasing under the experimental definition used in this study, stochastic augmentation occasionally generated local decreases that were physically inconsistent with cumulative-release behavior. Isotonic regression using the Pool-Adjacent-Violators Algorithm (PAVA) was therefore applied to enforce monotonicity while minimizing modification of the generated trajectories. The resulting monotonic profiles were subsequently used for kinetic characterization and predictive modeling.

#### 4.7.5. Weibull Kinetic Analysis

Each augmented release profile was independently characterized using the three-parameter Weibull model. The asymptotic cumulative release (*Q_∞_*), scale parameter (α), and shape parameter (β) were estimated using bounded nonlinear least-squares optimization. The corresponding half-release time (*t*_50_), representing the time required to reach 50% of the estimated asymptotic release, was calculated from the fitted Weibull parameters. Parameter distributions were summarized using the median and interquartile range (IQR).

#### 4.7.6. Comparative Evaluation of Release-Kinetic Models

To compare alternative empirical descriptions of the experimentally observed release behavior, mean cumulative-release profiles were fitted using zero-order, first-order, Higuchi, Korsmeyer–Peppas, and Weibull models. The Korsmeyer–Peppas model was restricted to the initial release phase (≤6 h) and was interpreted as an empirical descriptor of early release rather than as a mechanistic classifier. The remaining models were fitted across the complete experimental release profile.

Model performance was assessed using the coefficient of determination (R^2^), mean absolute error (MAE), root mean square error (RMSE), corrected Akaike Information Criterion (AICc), ΔAICc, and Akaike weights. Among the models fitted to the complete release profile, the model with the lowest AICc was considered to provide the most parsimonious description of the observed data. Because Korsmeyer–Peppas was fitted only to the restricted early-release interval, it was excluded from full-profile AICc-based model ranking.

#### 4.7.7. Early-to-Late Release Prediction

Early-to-late release predictability was evaluated using two complementary multi-output ridge-regression approaches. For both models, cumulative-release measurements obtained at 0.5, 1, 2, and 4 h were used as predictors together with compound identity encoded as a categorical variable. The first model directly predicted cumulative release at 6, 8, 24, and 48 h. The second predicted the Weibull parameters *Q_∞_*, α, and β, which were subsequently used to reconstruct complete release trajectories.

Before regression, *Q_∞_* was transformed using a logit transformation, whereas α and β were natural-log transformed and subsequently back-transformed for evaluation. The augmented dataset was divided into training and internal test subsets using an 80:20 stratified split while preserving representation of both compounds and augmentation strategies. Because both training and test profiles originated from the same experimental summary statistics, this partition was considered an internal assessment of predictive consistency within the augmented dataset rather than independent experimental validation.

Ridge regression was implemented with L2 regularization using λ = 1.0. Direct release predictions were evaluated using MAE, RMSE, and R^2^ at each prediction horizon, whereas Weibull-parameter predictions were evaluated using MAE and RMSE before reconstruction of the corresponding release curves.

#### 4.7.8. Exploratory Reconstructed-Replicate Holdout Assessment (LOBO-Style)

To complement augmented-data evaluation, an exploratory two-fold reconstructed-replicate holdout assessment was performed. Two release trajectories were mathematically reconstructed from the reported experimental means and standard deviations (n = 2), with higher and lower values assigned using the ordering of available raw HPLC peak areas. These trajectories represented reconstructed subsets of the original experimental dataset rather than independently prepared formulation batches. The uncertain β-sitosterol assignment at 0.5 h was additionally examined through sensitivity analysis.

In each holdout fold, one complete reconstructed batch trajectory was excluded from model development and used exclusively for evaluation, while the remaining reconstructed trajectory provided the training basis. Predictor variables comprised compound identity and cumulative release at 0.5, 1, 2, and 4 h, and ridge regression was used to predict cumulative release at 6, 24, and 48 h. The 8-h endpoint was excluded because its available value was regression-derived rather than independently available as a raw experimental replicate. Predictive performance was quantified using MAE, RMSE, and R^2^ at each horizon and across the pooled held-out predictions. This analysis was considered exploratory and was not treated as prospective external validation using independently generated formulation batches.

#### 4.7.9. Computational Environment and Reproducibility

All computational analyses were conducted in Python 3.12 using NumPy 2.1.3, pandas 2.2.3, SciPy 1.14.1, scikit-learn 1.5.2, and Matplotlib 3.9.2. Nonlinear least-squares fitting was implemented using the curve_fit function in SciPy, while monotonicity-constrained processing was performed using the IsotonicRegression implementation in scikit-learn. Stochastic procedures were initialized using a fixed random seed of 42 to ensure reproducibility of the data-augmentation and modeling workflow.

### 4.8. MTT Cell Viability Assay

The effects of free β-sitosterol, free naringenin, their free combination, and the corresponding single- and co-loaded nanophytosomal formulations on cell viability were evaluated using the MTT assay. Three human cell lines were examined: A549 (CCL-185™) lung adenocarcinoma cells, HCT116 (CCL-247™) colorectal carcinoma cells, and HEK293 (CRL-1573™) embryonic kidney cell lines were obtained from the American Type Culture Collection (ATCC; Manassas, VA, USA). The cytocompatibility of unloaded (blank) nanophytosomes was assessed during preliminary formulation development to evaluate potential carrier-associated cytotoxicity before investigating the bioactive-loaded formulations. Accordingly, the present comparative experiments focused on the cytotoxic responses associated with free and nanophytosomally encapsulated β-sitosterol and naringenin. Cells were maintained in complete growth medium supplemented with 10% fetal bovine serum and 1% penicillin–streptomycin at 37 °C in a humidified atmosphere containing 5% CO_2_.

Cells were seeded in 96-well plates at a density of 1 × 10^4^ cells per well in 100 µL of complete medium and allowed to attach overnight. The medium was subsequently replaced with fresh medium containing the respective treatments at concentrations spanning 6–200 µg/mL, and cells were incubated for 72 h. MTT solution was then added to each well, followed by incubation for 3–4 h at 37 °C to allow metabolically active cells to reduce MTT to insoluble formazan crystals. After removal of the supernatant, the resulting formazan crystals were dissolved in 100 µL dimethyl sulfoxide (DMSO), and absorbance was measured at 570 nm using 630 nm as the reference wavelength.

Cell viability was calculated relative to the corresponding untreated control and expressed as a percentage. Experiments were performed in triplicate, and results were reported as mean ± standard deviation (SD).

### 4.9. Molecular Docking Studies

Molecular docking was performed to evaluate the predicted interactions of β-sitosterol and naringenin with selected cancer-related molecular targets associated with the cellular models investigated in this study. Three experimentally resolved protein structures containing co-crystallized reference ligands were used to establish target-specific docking protocols. Protein and ligand preparation, receptor-grid generation, docking, redocking validation, pose-position assessment, and binding-site interaction analysis were performed using the Schrödinger molecular-modeling platform.

#### 4.9.1. Protein Structure Selection

Three protein structures were selected from the Protein Data Bank (PDB), each containing a co-crystallized inhibitor within the pharmacologically relevant binding site. MEK1 (*MAP2K1*) was represented by PDB 3EQC at 1.80 Å resolution, containing the allosteric inhibitor 3BM together with ATP-γ-S and magnesium. Human cyclooxygenase-2 (COX-2; *PTGS2*) was represented by PDB 5KIR at 2.70 Å resolution with rofecoxib as the reference ligand. Phosphoinositide 3-kinase α (PI3Kα; *PIK3CA*) was represented by PDB 4JPS at 2.20 Å resolution, containing the p110α/p85α complex with alpelisib bound. The structures and corresponding reference ligands are summarized in Table 11.

#### 4.9.2. Protein Preparation

Protein structures were prepared using the Schrödinger Protein Preparation Wizard. Bond orders were assigned, hydrogen atoms were added, missing side chains were completed, selenomethionine residues were converted to methionine, and disulfide bonds were generated where appropriate. N-linked and O-linked glycans in the 5KIR structure were retained and appropriately bonded. Ionization states of hetero groups were generated using Epik at pH 7.4, while residue protonation states were assigned using PROPKA at the same pH. Water molecules located more than 5 Å from hetero groups were removed. The prepared structures were subsequently minimized using the OPLS4 force field under heavy-atom restraints to a convergence threshold of 0.3 Å RMSD.

#### 4.9.3. Receptor and Reference-Ligand Definition

For each target, one copy of the co-crystallized reference ligand was extracted and retained as the experimental reference pose for subsequent redocking validation. Remaining copies of the reference ligand, residual water molecules, and crystallization additives, including thiocyanate, glycerol, sulfate, and polyethylene glycol, were removed from the receptor.

Catalytic or structurally relevant cofactors forming part of the binding environment were retained. ATP-γ-S together with magnesium, calcium, and sodium was retained in the MEK1 structure (3EQC), whereas cobalt protoporphyrin IX and the associated N-glycans were retained in the COX-2 structure (5KIR). Receptor composition was verified after preparation for each target.

#### 4.9.4. Receptor Grid Generation

Receptor grids were generated around the experimentally defined binding sites using the centroid of the corresponding co-crystallized reference ligand as the grid center. Primary docking grids were generated with an inner box of 12 Å and an outer box of 34 Å. The enlarged outer box was selected to accommodate the extended conformation of β-sitosterol, which spans approximately 20 Å, while permitting adequate orientational sampling within the binding site.

#### 4.9.5. Ligand Preparation

Naringenin and β-sitosterol structures were generated from their PubChem isomeric SMILES, with naringenin specified as the naturally occurring 2*S* enantiomer. Ligands were processed using LigPrep with Epik-based ionization and tautomer enumeration over pH 7.4 ± 2.0, the OPLS4 force field, and retention of specified stereochemistry. Naringenin generated five prepared states comprising the neutral form and several phenolate states, whereas β-sitosterol generated a single state.

For protocol validation, each co-crystallized reference ligand was independently extracted from its respective crystal structure and processed through the same LigPrep procedure. Consequently, redocking was performed using regenerated ligand conformers rather than the deposited crystallographic coordinates.

#### 4.9.6. Docking Procedure and Protocol Validation

Docking was performed using Glide in standard-precision (SP) mode. Identical docking settings were applied to each target-specific reference ligand and to β-sitosterol and naringenin. Ten poses were requested for each prepared ligand state, and ten poses were retained for post-docking minimization. The best-scoring minimized pose was selected for subsequent comparative analysis [26].

Each target-specific docking protocol was validated by redocking its corresponding co-crystallized reference ligand. Agreement between the top-ranked redocked pose and the crystallographic pose was quantified using symmetry-corrected heavy-atom root-mean-square deviation (RMSD), calculated in place without structural superposition. An RMSD ≤ 2.0 Å was predefined as the criterion for acceptable pose reproduction. Because crystallographic poses were available only for the reference ligands, RMSD was not calculated for β-sitosterol or naringenin. Instead, placement of the test compounds was quantified as the Euclidean distance between the centroid of the predicted pose and that of the corresponding crystallographic reference ligand.

For the MEK1 reference ligand 3BM, the distribution of pose deviation across the molecular structure was additionally examined by comparing the aromatic-core and non-ring atom subsets to distinguish displacement of the rigid pharmacophoric region from that of the flexible substituent.

For β-sitosterol at COX-2, failure to obtain a pose under the primary grid conditions was evaluated through a repeat docking run using an expanded inner box of 15 Å and outer box of 40 Å with expanded conformational sampling enabled. This repeat analysis was used to assess whether failure to generate a pose was attributable to the original grid dimensions or sampling conditions.

#### 4.9.7. Binding-Site Interaction Analysis

The best-scoring poses of β-sitosterol and naringenin were subjected to binding-site interaction analysis and compared with the corresponding reference ligands. Protein–ligand interactions were characterized using the Schrödinger Pose Interaction Analysis workflow and independently inspected using ligand-interaction diagrams generated in Maestro.

The analysis included hydrogen-bond interactions, hydrophobic contacts, aromatic and cation–π interactions where present, and contact-quality measures generated by the pose-interaction workflow. Interacting residues and hydrogen-bond distances were recorded for each ligand–target complex, together with the numbers of favorable, unfavorable, and severe contacts. These analyses were performed separately for PI3Kα, COX-2, and MEK1 to characterize target-specific interaction patterns and to support comparison of the test-compound poses with their respective crystallographic reference ligands.

### 4.10. Statistical Analysis

Quantitative experimental data were summarized as mean ± standard deviation (SD), with the number of independent replicates specified for each analysis. Statistical significance was defined at *p* < 0.05.

Particle size, polydispersity index (PDI), and zeta potential were compared among the naringenin-loaded, β-sitosterol-loaded, and co-loaded nanophytosomal formulations using one-way analysis of variance (ANOVA). Where appropriate, pairwise comparisons were performed using Tukey’s post hoc multiple-comparison test.

Encapsulation-efficiency and MTT cell-viability data were also analyzed inferentially, with comparisons performed between the relevant single/free and corresponding co-loaded or nanophytosomal conditions. Cell-viability analyses were conducted separately for A549, HCT116, and HEK293 cells, and statistical comparisons were evaluated at the corresponding treatment concentrations.

## 5. Conclusions

Although co-encapsulation is increasingly used in multicomponent nanocarriers, the formulation-specific consequences of jointly incorporating chemically distinct bioactives across encapsulation, release behavior, and biological response remain incompletely characterized. This study addressed this gap for β-sitosterol–naringenin co-loaded nanophytosomes by integrating physicochemical characterization, compound-specific release kinetics, machine learning-assisted prediction, cell-viability profiling, and molecular docking. Co-loading was associated with a marked increase in β-sitosterol encapsulation and a decrease in naringenin encapsulation, while the co-loaded formulation exhibited distinct compound-specific release profiles within the same phospholipid carrier. β-Sitosterol release was best described by the Weibull model, whereas naringenin followed Higuchi kinetics, demonstrating that co-encapsulated molecules can retain distinct release behavior despite sharing a common nanocarrier. Machine learning models further showed that later release could be predicted from the first 4 h of experimental measurements, providing a complementary data-driven dimension to conventional kinetic characterization.

These physicochemical differences were accompanied by cell-dependent biological responses. The co-loaded formulation showed its most favorable activity profile in A549 cells, with greater viability reduction than the free combination at submaximal concentrations while better preserving HEK293 viability; this advantage was not reproduced in HCT116 cells. Structure-based docking provided additional molecular context, with naringenin showing more favorable predicted accommodation across PI3Kα, COX-2, and MEK1, whereas β-sitosterol displayed weaker and target-dependent binding behavior.

Taken together, these findings show that co-loading β-sitosterol and naringenin can reshape their incorporation, release behavior, and biological responses within a shared nanophytosomal system. Integrating physicochemical characterization, kinetic modeling, machine learning prediction, cellular evaluation, and molecular docking provides a more comprehensive framework for assessing multicomponent nanocarriers. Further independent-batch validation, mechanistic and combination-effect analyses, and in vivo studies are warranted to establish the broader translational relevance of these findings.

## Figures and Tables

**Table 1:** Weibull kinetic parameters estimated from the augmented release populations of β-sitosterol and naringenin. Values are presented as median and interquartile range.

Compound	Q∞ (%)	α (h)	β	t50 (h)	R^2^
β-Sitosterol	86.60 (85.74–87.46)	1.43 (1.00–1.66)	3.23 (2.00–4.74)	1.18 (0.91–1.47)	0.994 (0.964–1.000)
Naringenin	70.58 (68.92–73.18)	8.40 (7.58–10.06)	0.52 (0.48–0.55)	4.19 (3.88–4.69)	0.935 (0.841–0.986)

**Table 2:** Comparative performance of empirical models fitted to the cumulative release profiles of β-sitosterol and naringenin.

Compound	Model	MAE (%)	RMSE (%)	R^2^	AICc	ΔAICc	Akaike Weight
**β-Sitosterol**	Zero-order	20.58	23.78	0.259	57.10	24.62	<0.001
	First-order	4.69	7.41	0.928	34.71	2.23	0.247
	Higuchi	17.98	20.74	0.436	54.91	22.43	<0.001
	Korsmeyer–Peppas *	10.63	13.51	0.781	—	—	—
	Weibull	3.75	5.10	0.966	32.48	0.00	0.753
**Naringenin**	Zero-order	2.72	4.31	0.938	29.76	3.39	0.155
	First-order	9.44	11.57	0.551	41.85	15.47	<0.001
	Higuchi	3.04	3.49	0.959	26.38	0.00	0.841
	Korsmeyer–Peppas *	0.23	0.28	0.991	—	—	—
	Weibull	6.42	6.86	0.843	37.20	10.82	0.004

* The Korsmeyer–Peppas model was fitted only to the initial release phase (≤6 h) and was therefore excluded from full-profile model comparison of the complete release profiles; consequently, AICc, ΔAICc, and Akaike weights are not reported.

**Table 3:** Internal test-set performance of the direct ridge-regression model for predicting later cumulative drug release from measurements obtained within the first 4 h.

Prediction Horizon (h)	MAE (%)	RMSE (%)	R^2^
**6**	1.23	1.57	0.996
**8**	1.74	2.09	0.992
**24**	1.00	1.17	0.992
**48**	1.00	1.25	0.976

**Table 4:** Predictive accuracy of the ridge-regression model for estimating Weibull kinetic parameters.

Weibull Parameter	MAE	RMSE
**Q∞ (%**)	0.910	1.145
**α (h**)	0.353	0.544
**β**	0.431	0.710

**Table 5:** Exploratory reconstructed-replicate holdout assessment for prediction of later cumulative release from early experimental release measurements.

Prediction Horizon (h)	MAE (%)	RMSE (%)	R^2^
6	4.95	6.99	0.916
24	2.51	3.50	0.917
48	2.49	2.71	0.853
Overall pooled	3.32	4.78	0.938

**Table 6:** Glide standard precision docking scores in kcal/mol and redocking validation. More negative scores indicate a better predicted fit. The per cent column expresses the naringenin score as a fraction of the validated reference score at the same target. The MEK1 value of 1.87 Å is measured over all heavy atoms; the aromatic core of the reference inhibitor redocks at 0.194 Å.

Target (Cell Model)	PDB	Reference Ligand	Reference Score	RMSD (Å)	Naringenin	β-Sitosterol	Ref. (%)
**MEK1 (A549**)	3EQC	3BM	−12.28	1.87	−7.61	−5.85	62
**COX-2 (A549**)	5KIR	Rofecoxib	−9.65	0.82	−8.40	No pose	87
**PI3Kα (HCT116**)	4JPS	Alpelisib	−10.81	0.36	−7.58	−5.45	70

**Table 7:** Distance between the centroid of each docked pose and the centroid of the crystallographic reference ligand.

Target	Reference Ligand	Naringenin	Beta-Sitosterol
MEK1, 3EQC	0.97 A	1.01 A	2.31 A
COX-2, 5KIR	0.25 A	0.72 A	No pose
PI3K alpha, 4JPS	0.22 A	2.50 A	0.82 A

**Table 8:** Binding-site interactions of the reference ligand, naringenin, and β-sitosterol at PI3Kα (PDB 4JPS). Hydrogen-bond distances are reported in Å, and contact counts were obtained from the Schrödinger pose-interaction analysis.

Ligand	Hydrogen Bonds	Hydrophobic Contacts	Good	Bad	Severe
**Alpelisib, reference**	Gln859 (two), Val851 backbone (two), Ser854, aromatic to Trp780	Tyr836, Ile848, Ile932, Ile800, Val851, Met922	357	6	1
**Naringenin**	Val851 backbone NH 2.03 A and C=O 1.97 A, Asp933 1.96 A	Ile800, Ile848, Trp780, Met922, Ile932	223	4	0
**beta-sitosterol**	Asp933 only, 1.70 A	Met922, Trp780, Val850, Ile800, Ile932, Ile848, Met772, Arg852	424	42	4

**Table 9:** Binding-site interactions of rofecoxib and naringenin at COX-2 (PDB 5KIR). β-Sitosterol yielded no docked pose under the evaluated conditions.

Ligand	Hydrogen Bonds	Hydrophobic Contacts	Good	Bad	Severe
Rofecoxib, reference	Arg513 1.78 A, aromatic to Met522	Val349, Ala527, Leu352, Trp387, Val523, Phe518, Ala516	335	5	1
Naringenin	Ser530 1.61 A, Gln192 1.98 A, His90 2.23 A, Tyr355	Val523, Phe518, Leu352, Trp387, Val349	285	10	1
beta-sitosterol	No pose obtained	No pose obtained	n/a	n/a	n/a

**Table 10:** Binding-site interactions of the reference inhibitor 3BM, naringenin, and β-sitosterol at MEK1 (PDB 3EQC).

Ligand	Hydrogen Bonds	Good	Bad	Severe
3BM, reference	Lys97, Asp190, ATP-gamma-S O2G, aromatic to Phe209	332	10	2
Naringenin	ATP-gamma-S O3G 1.35 A and O2G 2.59 A, Ser212, cation-pi to Lys97; no direct side chain hydrogen bond in the contact analysis	270	13	1
beta-sitosterol	3-hydroxyl to Arg234 in the interaction diagram, none in the contact analysis	500	38	2

**Table 11:** Crystal structures and reference ligands used for molecular docking.

Target	Gene	Cellular Context	PDB	Resolution (Å)	Reference Ligand
MEK1	*MAP2K1*	A549	3EQC	1.80	3BM
COX-2	*PTGS2*	A549	5KIR	2.70	Rofecoxib
PI3Kα	*PIK3CA*	HCT116	4JPS	2.20	Alpelisib

## Data Availability

The raw data supporting the findings of this study are provided within the main text.

## Abbreviations

AICc	Corrected Akaike information criterion
ANOVA	Analysis of variance
ATP-γ-S	Adenosine 5′-O-(3-thiotriphosphate)
COX-2	Cyclooxygenase-2
DLS	Dynamic light scattering
DMSO	Dimethyl sulfoxide
DSPC	Distearoylphosphatidylcholine
EE	Encapsulation efficiency
HPLC	High-performance liquid chromatography
IQR	Interquartile range
LOBO	Leave-one-batch-out
MAE	Mean absolute error
*MAP2K1*	Mitogen-activated protein kinase kinase 1
MEK1	Mitogen-activated protein kinase kinase 1
MTT	3-(4,5-Dimethylthiazol-2-yl)-2,5-diphenyltetrazolium bromide
OPLS4	Optimized Potentials for Liquid Simulations 4
PAVA	Pool-Adjacent-Violators Algorithm
PBS	Phosphate-buffered saline
PDB	Protein Data Bank
PDI	Polydispersity index
PEG	Polyethylene glycol
PI3Kα	Phosphoinositide 3-kinase alpha
*PIK3CA*	Phosphatidylinositol-4,5-bisphosphate 3-kinase catalytic subunit alpha
*PTGS2*	Prostaglandin-endoperoxide synthase 2
R^2^	Coefficient of determination
RMSD	Root-mean-square deviation
RMSE	Root-mean-square error
SD	Standard deviation
SMILES	Simplified Molecular Input Line Entry System
SP	Standard precision
ΔAICc	Difference in AICc relative to the best-supported model
